# An Integrated MODEL-OPTI-SCALE Computer System for Modeling, Optimization, and Scaling-Up of Plastic Extrusion

**DOI:** 10.3390/ma19173744

**Published:** 2026-09-03

**Authors:** Krzysztof Wilczyński, Andrzej Nastaj

**Affiliations:** The Faculty of Mechanical and Industrial Engineering, Polymer Processing Institute, Warsaw University of Technology, 02-524 Warsaw, Poland; andrzej.nastaj@pw.edu.pl

**Keywords:** plastic extrusion, process modeling, optimization, scaling-up

## Abstract

An integrated Model-Opti-Scale (MOPS) computer system for the modeling, optimization, and scaling-up of plastic extrusion has been built. This enabled simulations of single-screw extrusion with conventional, gravitational feeding of the extruder and single-screw extrusion with metered feeding. This system enables predictions of the behavior of these processes using material, geometry, and operating data and contains numerous original and unique models and algorithmic solutions. The system co-operates with specially developed optimization and scaling procedures based on Genetic Algorithms, allowing process optimization/scaling-up. A unique, holistic approach to modeling flood-fed/starve-fed processes, with a possibility of transfer between these feed methods, is presented. The problem of optimizing the operating/geometry parameters of single-screw extrusion for maximizing process efficiency and minimizing specific energy use has been solved. Optimization has been achieved in a unique way, allowing the coupling of both methods of feeding. It has also been observed that an optimal solution may be obtained upon flood feeding or starve feeding, depending on the assumed weights of the optimization criteria. This optimized process was scaled up to increase process efficiency by enlarging the extruder diameter while keeping the length/diameter ratio constant. Scaling-up has been achieved using criteria comprising single parameters, including specific energy consumption, material temperature, and the plasticization rate, as well as criteria comprising functional parameters, such as temperature distribution and the material plasticization profile.

## 1. Introduction

Extrusion is, next to injection molding, the most important plastics processing technique. It is also the most common mass-production technique used to produce various types of profiles, as well as pipes, films, etc. It is also applied in compounding processes.

Extrusion can be carried out in single-screw or twin-screw systems, co-rotating or counter-rotating. These can be flood-fed (with gravitational feeding) or starve-fed (with metered feeding), i.e., with material dosing. Dosing is typically used in twin-screw extruders.

**Figure 1:**
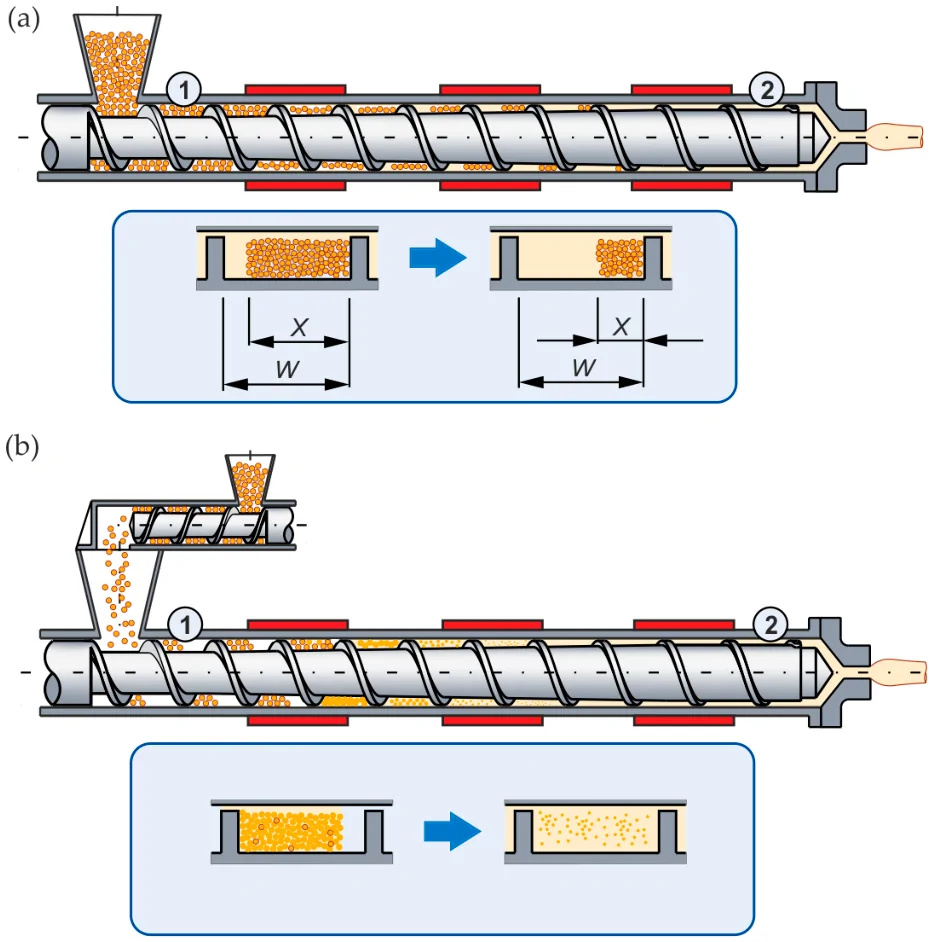
Plasticization in single-screw extrusion: (**a**) flood feeding, (**b**) starve feeding, (1) solid transport region, and (2) melt transport region. X—solid bed width; W—channel width.

In gravitational feeding, the screw draws in as much plastic as it can handle and is thus fully filled. In dosed feeding, the plastic is fed into the machine via a feeder, and the screw is not fully filled with plastic (Figure 1).

The first extruders were ram machines. Screw machines appeared in the 1860s. The literature on extrusion is very extensive, and many books and chapters have been written on this topic, e.g., [1,2,3,4,5,6,7,8,9,10,11,12,13,14,15,16,17,18,19,20,21,22,23,24,25]. What is interesting is that groundbreaking work in extrusion occurred in the last century, e.g., [2,3,5,7,9,10], and at the beginning of this century, e.g., [14,15,18,21,22,23].

The design of plastics processing is currently supported by process simulations. These enable prediction of the process behavior by using material, geometry, and operating data. For example, extrusion can be described by its flow rate, temperature, pressure distribution, energy use, mixing efficiency, etc. This means that output process data can be predicted when using input data.

The first description of material transport in screw machines (screw pump), both isothermal and Newtonian, was given by Finlayson and Rowell [26] for viscous oils. This was reconsidered by Carley’s group [27], who developed the first model of plastics flow in an extruder. Later, many researchers improved these basic models by developing 3D, non-Newtonian, and non-isothermal ones in an actual screw geometry, considering mechanical/thermal coupling, e.g., [28,29,30].

Solid transport in an extruder was considered first by Mol and Darnell [31]. The solid pellets were assumed to be compacted into a solid bed, which moves due to friction on the barrel (cylinder) and screw. Later, other researchers extended this approach, e.g., [32,33]; however, the basic analysis remained unchanged. Some investigations, e.g., [34], showed that basic assumptions are no longer valid since relative movement of pellets takes place in the extruder. Thus, the discrete element method (DEM) was taken into account. This research concept is beneficial in that each pellet and its interaction with others can be modeled individually. However, higher computing time is an important drawback.

Basic 3D DEM simulations of compacting pellets in single-screw machines were carried out by Thompson and Moysey [35,36]. Research by Schöppner et al. [37] has been performed, considering axial pressure development in the feed region of single-screw machines. The DEM concept has also been applied to model solids transport in twin-screw machines [38]. However, so far, this method has not been applied to extrusion global modeling since it is very time-consuming. First, tests of material plasticization in an extruder were performed by Maddock and Street [39,40]. An analysis of a material removed from a screw allowed recognition of the plasticization process. It was observed that a melt film appears on the solid surface, which is scraped off and accumulates at the active screw flight (Figure 1a). Plasticization progresses; the solid decreases by heat conduction from the cylinder and viscous dissipation in the molten plastic.

Tadmor et al. [41,42] developed the first plasticization model for single-screw extrusion and formulated the first global model of the process, called EXTRUD [43]. Others extended the work of Tadmor, e.g., [44,45,46], but the basic analysis remained unchanged. Next, several computer models of single-screw extrusion have been built, e.g., NEXTRUCAD by Vlachopoulos and Agur [47], PASS by Rakos and Sebastian [48], REX by Potente et al. [49], and SSEM by Wilczyński [50], who extended the first basic models of neat polymer extrusion with conventional screws to advanced models of extrusion with non-conventional screws [51,52], and extrusion of polymer blends [53] as well as polymer composites [54,55,56]. Valuable models were also developed by Nomura et al. [57], Karnis and Zavadsky [58], and Lafleur et al. [59].

Twin-screw processing is much less researched than single-screw processing. Co-rotating twin-screw processing was first studied by White’s group. Based on melt and plasticization studies [60,61,62,63], they built the first model, called AKRO-CO-TWIN [62]. Independent studies were performed by the Potente group [64,65], who developed the SIGMA program [66], and by the Vergnes group, who developed the LUDOVIC program [67]. A counter-rotating twin-screw process was also first studied by White’s group. Based on the melt and plasticization studies [68,69,70,71], they built the first model, called AKRO-COUNTER-TWIN [72]. This was continued by Wilczyński’s group, who developed the TSEM (Twin-Screw Extrusion Model) program [73,74,75,76,77].

Although the flood-fed process was extensively investigated, little information was presented on the starve-fed process [78]. Wilczyński et al. [79], based on experiments, first proposed the plasticization model for this process [80]. According to this model, two phases of plasticization were distinguished: plasticization by conduction in the partially filled region of the screw, and plasticization by energy dissipation in the fully filled region, where the pellets are dispersed in molten material (Figure 1b). Based on this, the first and only currently available model of the process was built [81,82]. This was later extended to non-conventional screws [52,83] and extrusion of polymer blends [84,85], as well as polymer composites [86,87,88,89].

It is worth noting that the starve-fed process has some benefits over the flood-fed process, e.g., [22,78,90,91,92,93]. Generally, starvation improves plasticization and mixing actions, reduces power/energy consumption, and results in a small reduction in extrusion output.

One issue with plastics extrusion modeling was discussed in fundamental monographs, e.g., by White and Potente [14], Rauwendaal [22], and the Agassant group. [23], and in review articles, e.g., by Ariffin et al. [94], Teixeira et al. [95], Malik et al. [96], and Wilczyński et al. [97,98].

**Figure 2:**
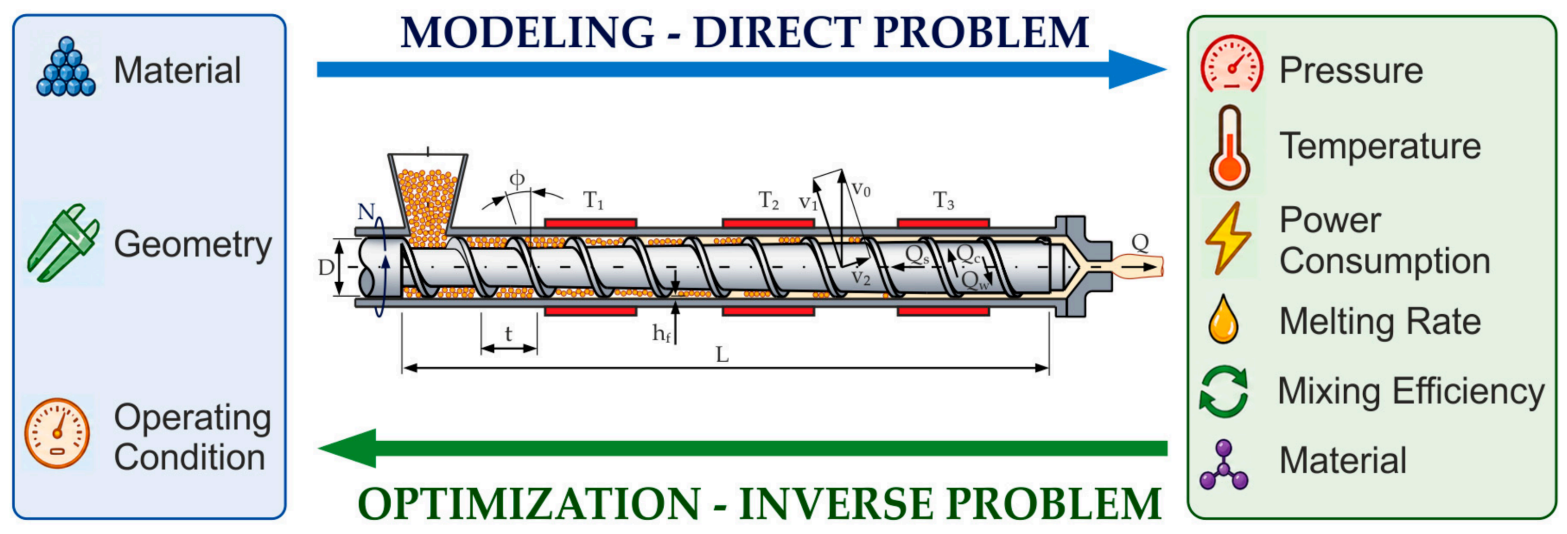
Direct (modeling/simulations) and inverse issues (optimization) regarding plastics extrusion: L, D, t, φ, h—geometry data; N—screw speed; Q—throughput; T—temperature data; V—screw circumferential velocity.

Computer modeling is useful in process design; however, it does not allow solving an inverse problem of defining the process input variables to obtain optimal output variables. Thus, this does not enable optimization of the process in terms of specific optimization criteria. Modeling (simulations) is a direct issue, while optimization is an inverse issue (Figure 2).

There are two concepts for solving the optimization issue: experiments or simulations. Experiments come first; the optimum was simply the best of what had been attempted. However, it was time-consuming, expensive, and there was no guarantee of finding a global optimum.

Underwood [99] and Verbraak and Meijer [100] were the first to use DOEs (Design of Experiments) to optimize the extrusion process. The optima were found by searching the extremes on the response surface relating to the input/output variables. The main drawback of this was the number of experiments needed.

Therefore, using simulations seemed to be more effective. First, optimization via simulation was performed by Tadmor and Klein [5], Maddock and Smith [101], and Helmy and Parnaby [102]. Potente et al. developed a more sophisticated strategy for screw optimization by means of DOEs (Design of Experiments), using the extrusion model REX [49,103]. Lafleur and Thibodeau used their own extrusion model [59,104]. Statistical procedures were also applied by the authors using SSEM (Single-Screw Extrusion Model) software [51]. The main disadvantages of using statistics were the number of simulations needed and the danger of finding local optima rather than a global one.

Artificial Intelligence (AI), e.g., Genetic Algorithms (GA), Neural Networks (NN), and Fuzzy Systems (FS), can also be used to optimize the extrusion process. Basic investigations with Genetic Algorithms were conducted by Covas and Gaspar-Cunha, who were the first to elaborate the optimization algorithms for the single-screw [105,106] and co-rotating twin-screw processes [107,108]. The authors proposed optimization by using Neural Networks and Genetic Algorithms for the single-screw process, both flood-fed and starve-fed [109], as well as for the counter-rotating twin-screw process [110]. However, an issue of optimizing the extrusion process has been recently summarized by Nastaj and Wilczyński [111].

Genetic Algorithms, compared to other optimization techniques, differ in the following manner:

- Optimization variables are used in coded form;
- Seeking a solution starts from some populations; therefore, becoming stuck in a local extremum is less likely;
- A new search area is based on previous experiences;
- Selections are probabilistic;
- The objective function is needed, not its derivatives.

**Figure 3:**
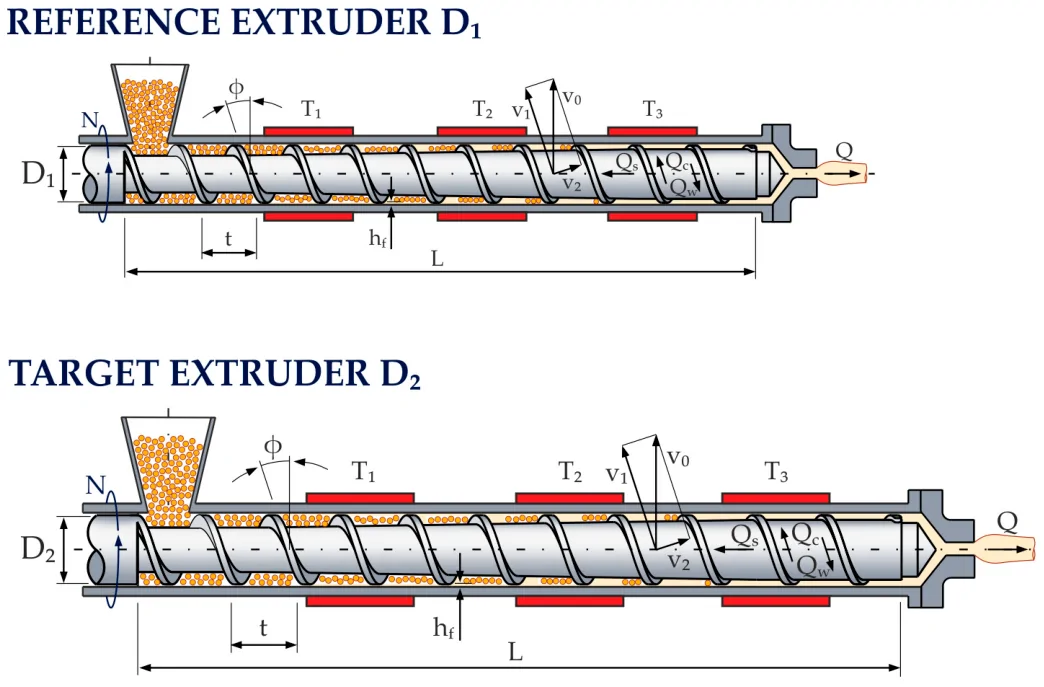
Scaling-up in plastics extrusion: D_1_—reference screw; D_2_—target screw.

Another concept of designing in plastics extrusion is what is known as scaling-up, i.e., increasing the scale of the process by using specific criteria, such as increasing process efficiency while keeping the scaling variables as close (similar) to the reference process as possible. Thus, this consists of minimizing the differences between the variables of the reference/target processes. This concept results from the theory of similarity in fluid mechanics. The scaling-up concept for plastics extrusion is shown in Figure 3.

McKelvey and Carley [112] were the first to carry out the scaling of extrusion. They developed scaling-up rules for screws in which the channel depth/width were enlarged in proportion to the screw-to-diameter ratio at a constant screw speed. Other scaling-up concepts were later developed [113,114,115,116,117,118,119,120,121], among which Pearson [115] was the first to perform a full analysis of the process, considering solid conveying, material plasticization, and melt conveying. He achieved acceptable scaling-up when the numbers of Brinkman, Graetz, and Nahme were kept constant.

Covas and Cunha [122] concluded that more effective scaling-up concepts are necessary and should be based on the model of the process that enables the simultaneous application of several scaling-up criteria, as well as the selection of single parameters (e.g., plasticization rate) or functions (e.g., plasticization progress along the screw) as scaling-up criteria.

They proposed considering extrusion scaling as a multi-objective optimization issue, where the goal is to define the geometry/operation data of the target extruder in such a way that the performance data of both extruders are as close (similar) as possible.

**Figure 4:**
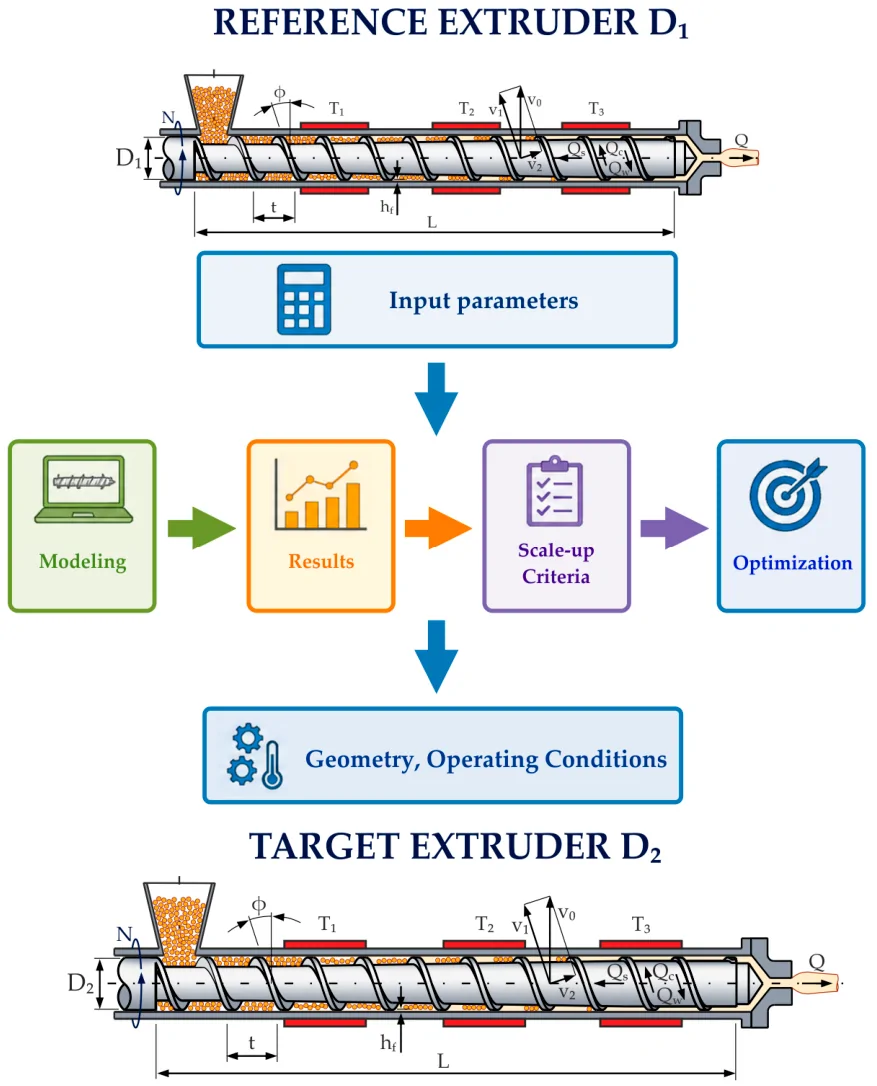
The optimization concept of the scaling-up of plastics extrusion process: D_1_—reference screw; D_2_—target screw.

The goal of scaling is to minimize the differences between selected process variables of the target/reference extruders. This concept involves process modeling/simulations, defining the scaling criteria, and multi-objective optimization. The scaling criteria are computed for the target process and compared with the criteria of the reference one, and each computation is evaluated. Searching for a good solution is repeated until a satisfactory minimization of the differences is obtained. This concept is presented in Figure 4.

Using this multi-objective optimization concept [105,106,107,108], Covas and Cunha [122] were the first to perform comprehensive scaling-up of the conventional flood-fed single-screw process in terms of screw geometry and operating data. This concept was later applied to various scaling-up issues of the single-screw and co-rotating twin-screw processes [123,124,125,126,127]. Recently, the authors developed scaling-up algorithms for the starve-fed single-screw [128] and counter-rotating twin-screw processes [129]. The state-of-the-art in this area has been recently presented in the review article [111].

It can be concluded that using process simulations seems to be a fundamental and most effective concept for both optimizing and scaling up plastics extrusion.

As evidenced by the presented literature review, modeling, optimization, and scaling are subjects of research and scientific debate. However, a system that would enable an integrated design of the extrusion process by using these design techniques is lacking. There is also a lack of a system that would enable smooth transitions between different extrusion techniques, such as extrusion with material dosing and extrusion without material dosing. This paper presents such a system that, based on a holistic approach to design, addresses these issues. In this paper, using this system, the design of single-screw extrusion based on process modeling, optimization, and scaling up has been performed.

## 2. Model-Opti-Scale System

The Model-Opti-Scale (MOPS) system is composed of three closely related and co-operating subsystems: GSEM (Global Screw Extrusion Model) for simulation of the single- screw process, GASEO (Genetic Algorithms Screw Extrusion Optimization) for process optimization, and GASES (Genetic Algorithms Screw Extrusion Scaling) for process scaling. The system enables the design of the extrusion process based on process modeling, optimization, and scaling, in dosed and non-dosed feeding conditions (starve-fed/flood-fed), with the possibility of transfer between these feed methods.

### 2.1. Process Modeling

Process modeling was carried out by the GSEM (Global Screw Extrusion Model) program, which has been designed to simulate the single-screw process, both flood-fed and starve-fed. This enables simulations of the process with the use of conventional/non-conventional screws and dies of various geometries. The program simulates the extruder/die system, enabling prediction of the flow rate (when flood-fed), screw filling (when metered-fed), temperature and pressure profiles over the extruder/die length, plasticization of material, and energy use, etc. The simulation data include material characteristics, screw/die geometry, and operating conditions.

The global model of the extrusion process consists of elementary screw models and a die model. The polymer mass flow rate is constant in screw/die increments, and the process parameters may be assumed locally constant in these increments. This modeling approach is called lumped parameter modeling, which is accurate enough for engineering practice and applications. The parameters of the extrusion process, e.g., flow rate, temperature, power, pressure, screw filling, at the onset of the current screw increment are equal to the parameters at the exit of the former increment, i.e.,

(1) f_i−1_out_ (z) = f_i_in_ (z)

(2) f_i−1_out_ (z + Δz) = f_i_in_ (z + Δz)

where f_i_in_ (z) is the input data (temperature, pressure, or solid material content) at the onset of the increment; f_i−1_out_ (z) is the output data at the exit of the (i − 1) increment; z is the coordinate along the screw/die axis; and Δz is the screw/die increment.

Summarizing, the model consists of elementary models, depending on the material being processed (pure polymers, polymer blends, WPC):

- Model of the solid conveying section;
- Model of the melting section;
- Model of the screw melt conveying section using the three-dimensional, non-Newtonian flow characteristics;
- Model of the die-flow section using the three-dimensional, non-Newtonian flow characteristics.

**Figure 5:**
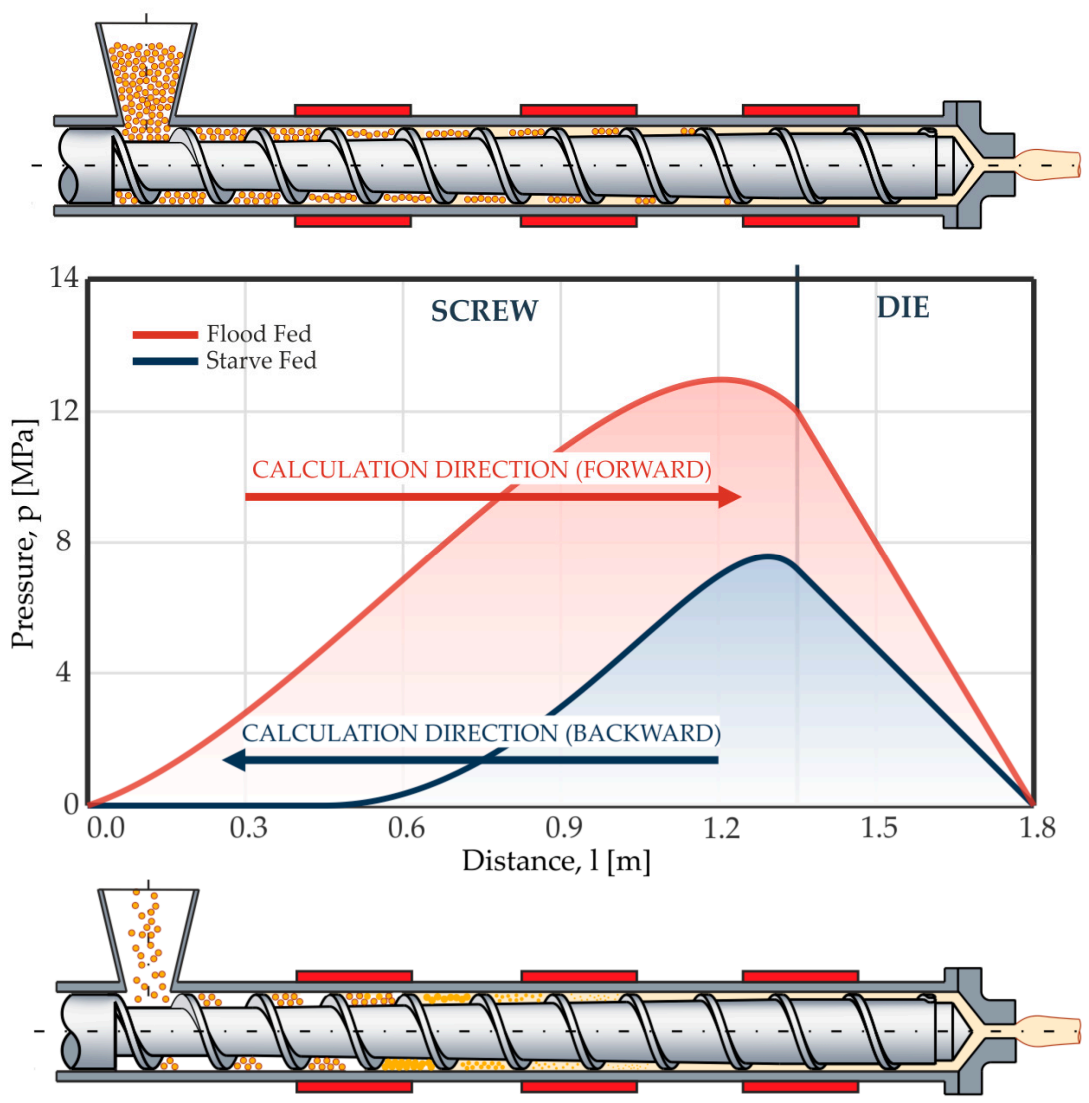
Forward algorithm for flood-fed process vs. backward algorithm for metered-fed process.

Extrusion modeling requires a computation algorithm that is appropriate to the type of extruder feeding. In the GSEM program, when flood-feeding, the forward algorithm is used. The material flow rate is not known here, and the results from the extruder/die operation have to be calculated in multiple iterative computations. When metered-feeding, the backward algorithm is used. The flow rate is known here, and it is equal to the flow rate of the material supplied by the dosing instrument (Figure 5).

**Figure 6:**
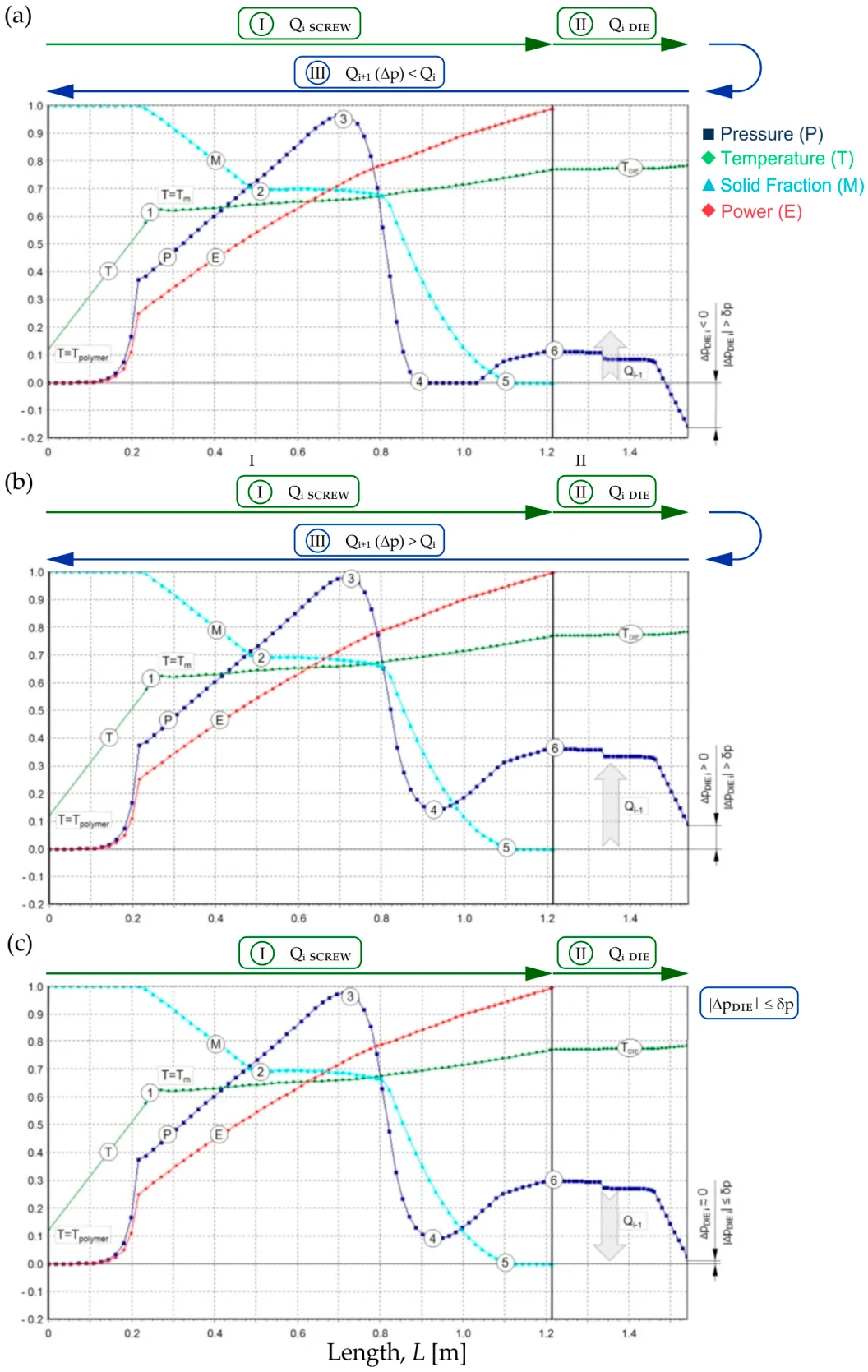
Course of simulation of the flood-fed process: (**a**) Δp_die_ < 0; (**b**) Δp_die_ > 0; (**c**) ׀Δp_die_׀ < δp. 1—origin of material plasticization; 2—origin of compression (transition) region; 3—pressure maximum; 4—finished pressure drop; 5—finished material plasticization; 6—pressure at screw outlet (die inlet); Q—flow rate; Q_i+1_—next flow rate value; P—pressure; Δp_DIE_—pressure at die outlet; δp—pressure calculation accuracy; T—temperature; T_m_—melting point; T_DIE_—melt temperature (die region); M—solid fraction (plasticization), E—power.

The course of simulation of the flood-fed extrusion is presented in Figure 6. The modeling progresses from the hopper to the die (forward algorithm), and the process operating point is sought, which determines the process output and die pressure. Calculations start with a presumed flow rate, and then solid transport, material plasticization, and melt transport are simulated. The pressure at the die outlet is compared to atmospheric pressure, and the calculation is correct when both pressures are equal. Otherwise, the flow rate is varied, and the calculation is repeated until convergence is obtained.

**Figure 7:**
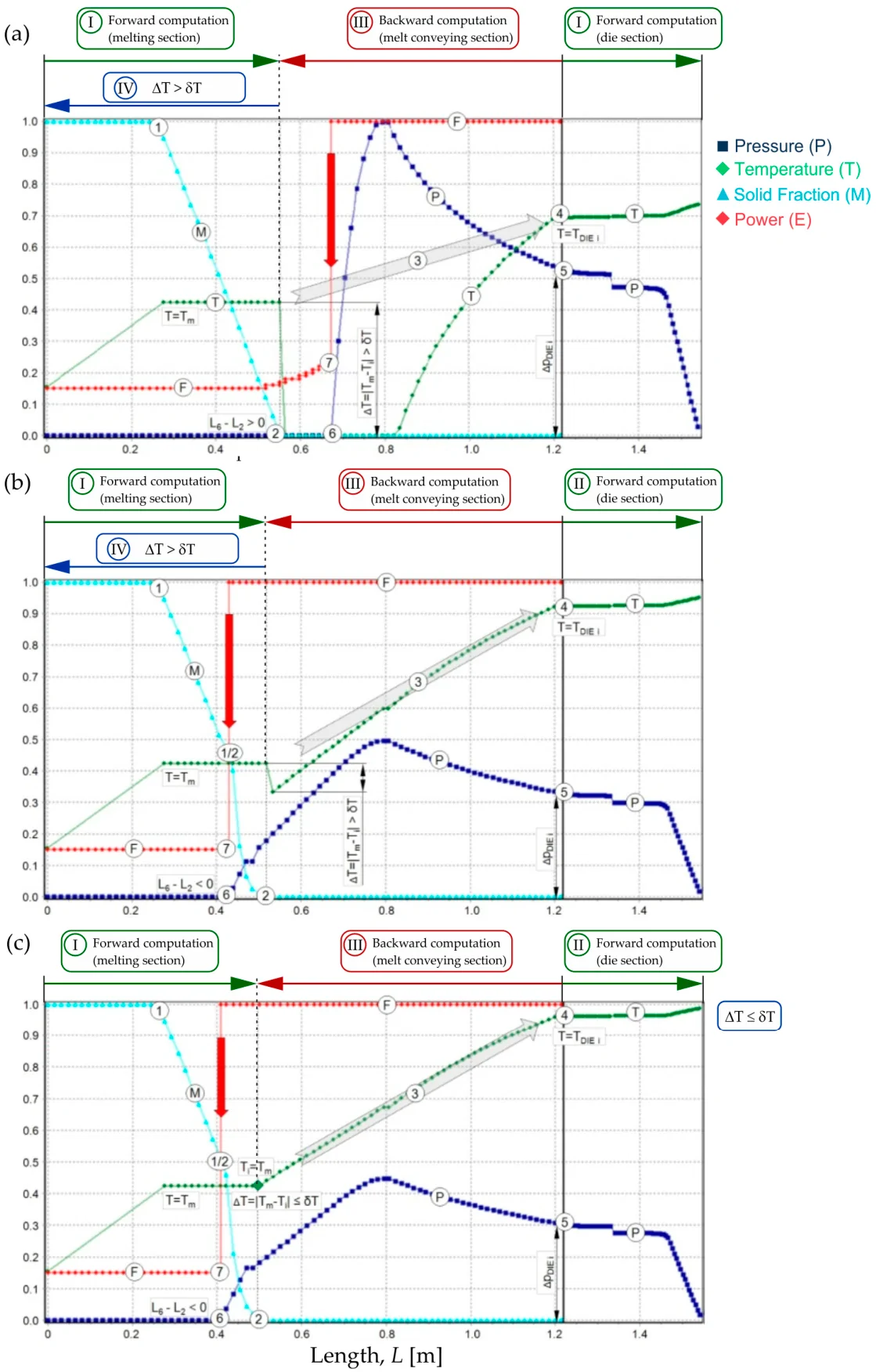
Course of simulation of the starve-fed extrusion: (**a**) 1-phase plasticization mechanism, lack of calculation convergence; (**b**) 2-phase plasticization mechanism, lack of calculation convergence; (**c**) calculation convergence. I—forward calculations (melting region); II—forward calculations (die region); III—backward calculations (melt conveying region); IV—next iteration; M—solid fraction (plasticization); F—filling (fill factor); T—temperature; P—pressure; 1—origin of material plasticization; 2—finished material plasticization; 3—transfer of calculations to the die; 4—origin of melt temperature calculations; 5—origin of die pressure calculations; 6—zero pressure location; 7—origin of filling calculations (partially filled region starts); ΔP_DIEi_—die pressure; T_DIEi_—presumed melt temperature; T_m_—melting point; i—number of iterations; ΔT = |T_m_ − T_i_|, convergency testing; δT—temperature computation accuracy.

The course of simulation of the starve-fed process is presented in Figure 7. In this case, the backward algorithm of computation is used. The flow rate is equal to the feeding rate, and the die pressure is calculated first for a presumed melt temperature. The pressure gradient over the screw length is calculated (as well as the temperature) with the use of screw-pumping characteristics. When the pressure diminishes to zero, the starvation origins and the screw filling (filling factor) are calculated. The temperature at the plasticization end is compared to the melting point, and the calculation is correct when both temperatures are equal. Otherwise, the melt temperature is varied, and calculations are repeated until convergence is obtained.

**Figure 8:**
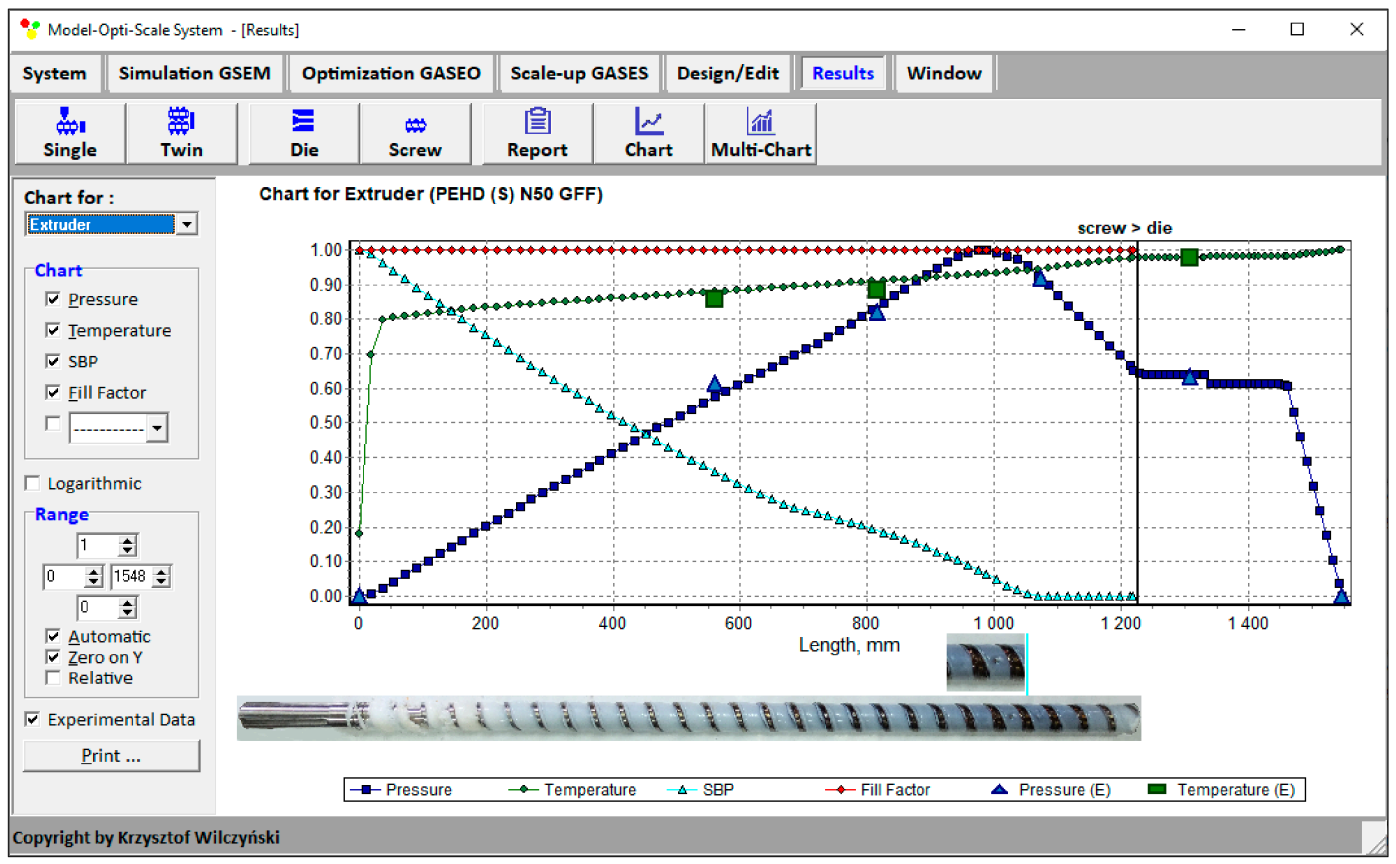
Extrusion process characteristics: simulations and experimental data for flood-fed extrusion: SBP—solid bed profile (material plasticization); E—experiment.

**Figure 9:**
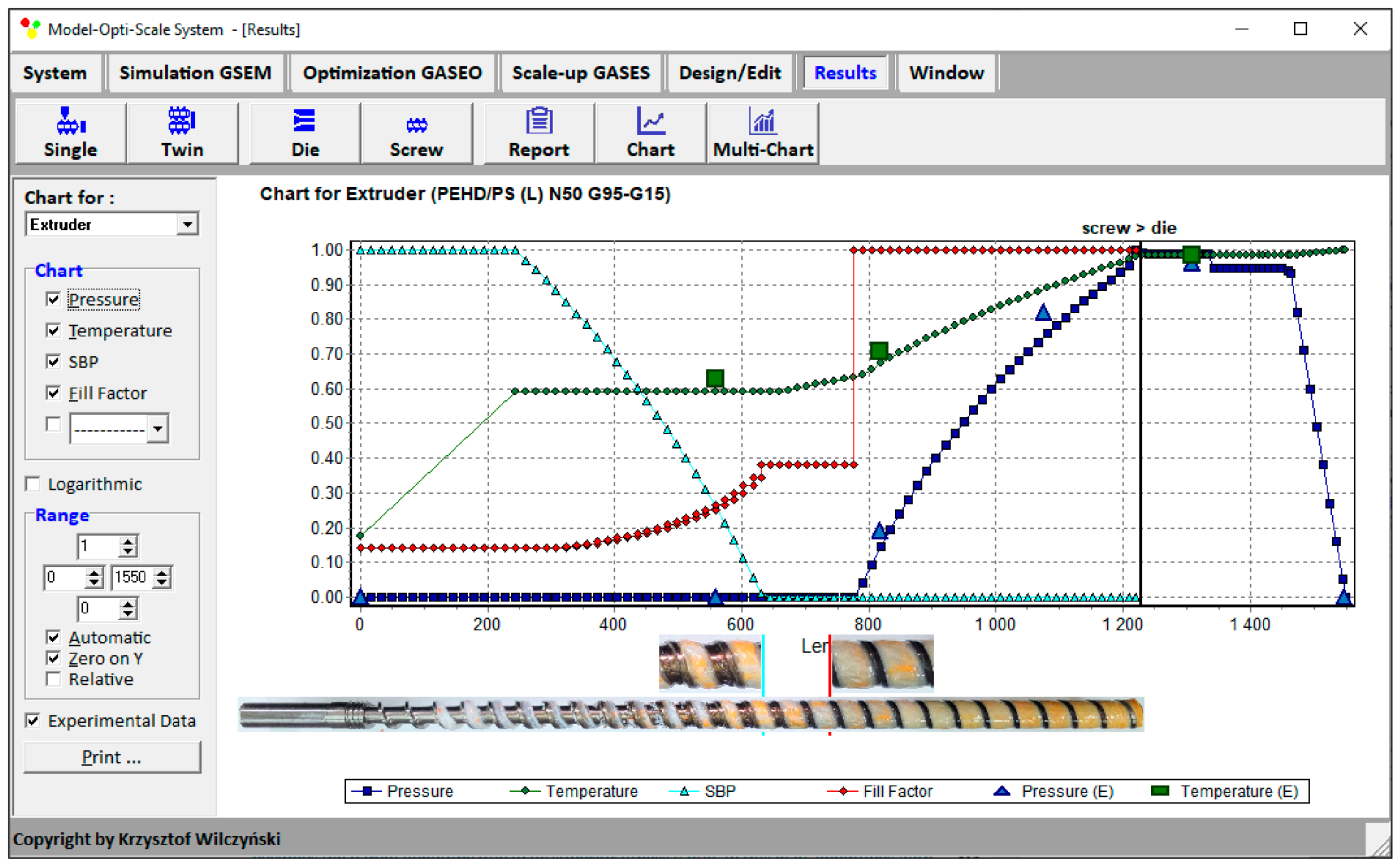
Extrusion process characteristics: simulations and experimental data for starve-fed extrusion: SBP—solid bed profile (material plasticization); E—experiment.

The simulation examples are shown in Figure 8 and Figure 9. The process characteristics include pressure/temperature distributions, as well as material plasticization progress (solid bed profile, SBP). In addition, for starve-fed extrusion (Figure 9), a screw filling profile is presented (when starvation begins, the pressure diminishes to zero). Two phases of plasticization are seen, as well as the partially filled and fully filled regions.

These simulations (by the GSEM model) were experimentally validated in the authors’ previous studies, and the simulation–experiment comparisons shown in Figure 8 and Figure 9 provide an illustrative confirmation of its predictive capability. In contrast, the optimization and scale-up results presented in the following sections constitute computational demonstrations of the corresponding MOPS modules.

### 2.2. Process Optimization

Process optimization was carried out by the GASEO (Genetic Algorithm Screw Extrusion Optimization) program. The source of data for optimization is the simulations carried out by the GSEM (Global Screw Extrusion Model) program.

**Figure 10:**
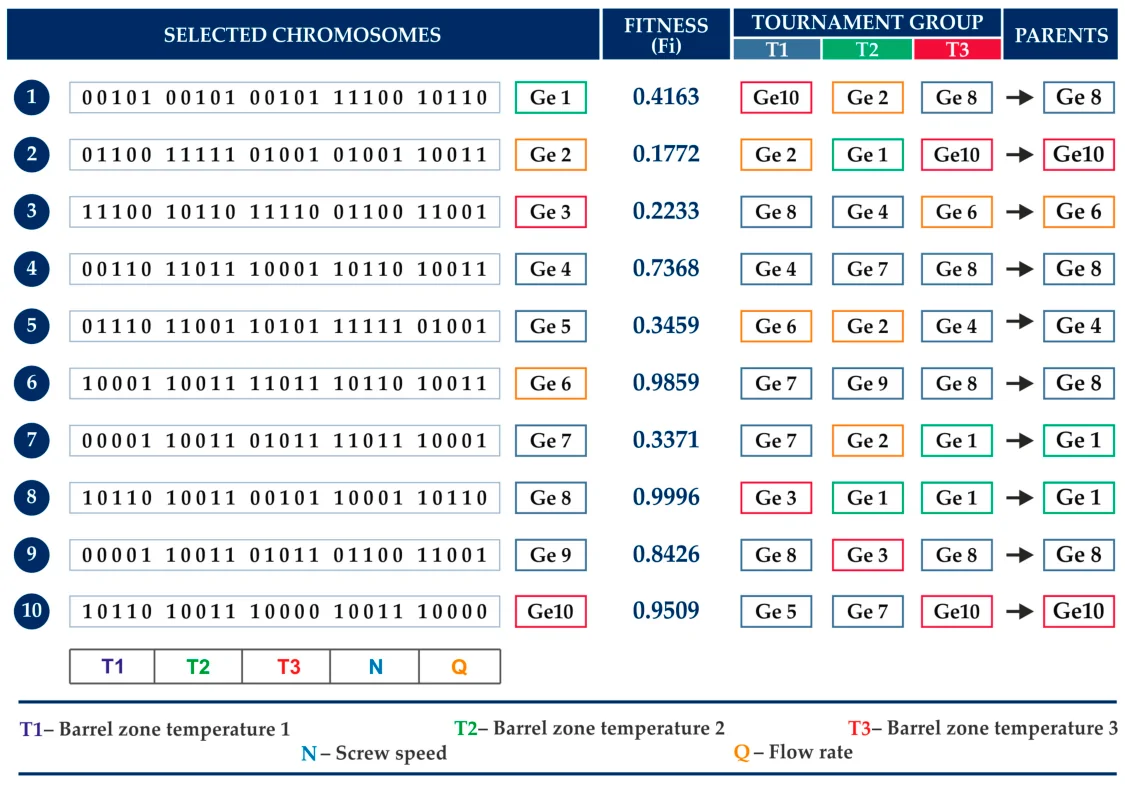
Selection of initial population and evaluation of chromosome adaptation.

The GASEO optimization program, in co-operation with the GSEM simulation program, enables extrusion process optimization with any number of variables and various optimization criteria, e.g., process output or energy use. The accuracy of searching the response surface is defined by the number of divisions of the variable range, which results from the binary representation of variables. In the GASEO program, the binary chromosome length is adjustable, with a maximum length of 255 bits. This allows the range of each variable to be divided into 2^255^ values. A tournament selection method is used for parent selection because of its simplicity, robustness, and lower sensitivity to scaling the objective function. In this method, a group of randomly selected genotypes participates in a tournament, and the genotype characterized by the highest objective function value is selected as a parent. The procedure is repeated until the required number of parents is obtained. A scheme of the tournament selection method is presented in Figure 10. For example, in a tournament composed of genotypes Ge2, Ge10, and Ge8 (first line), the Ge8 genotype is selected because it generates the highest objective function value. Similarly, in another tournament consisting of Ge2, Ge1, and Ge10 (second line), the Ge10 genotype becomes the parent genotype due to its superior fitness value. In this way, genotypes with higher objective function values have a greater probability of being selected for reproduction while preserving population diversity.

**Figure 11:**
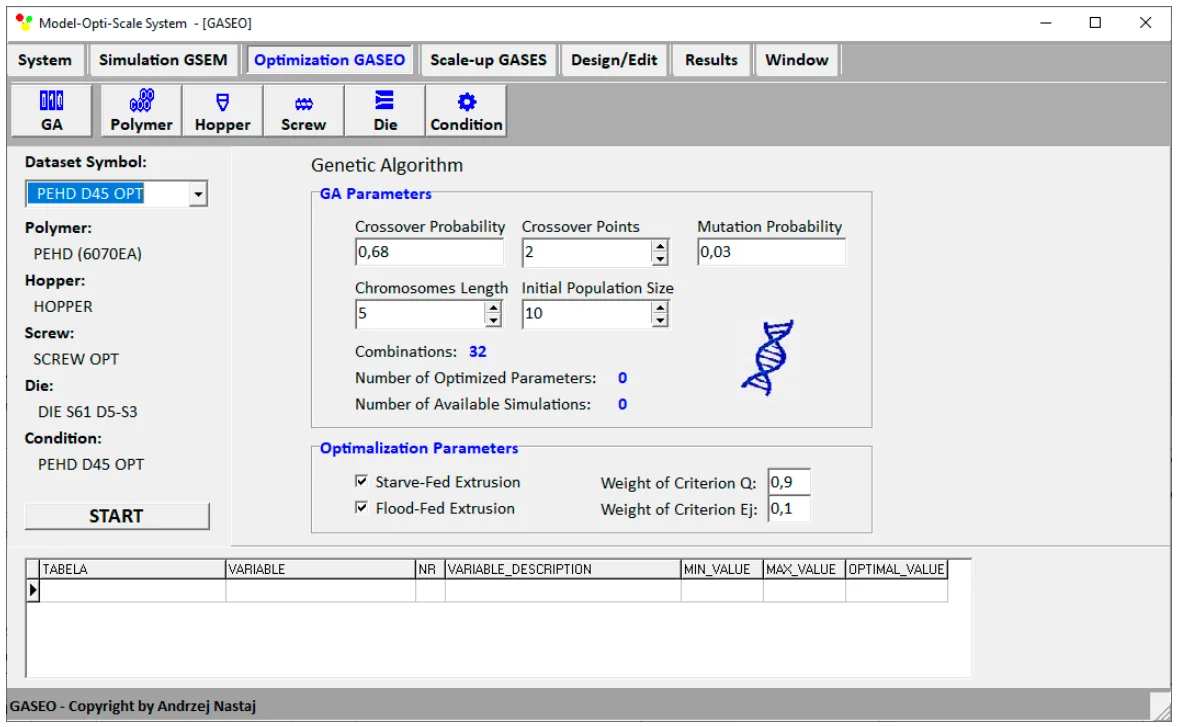
Parameters defining the optimization procedure.

The optimization procedure was defined by Genetic Algorithm variables, including the number of optimized variables, population size, chromosome length, crossover probability, number of crossover points, and mutation probability. Optimization can be carried out selectively for either the flood-fed process or starve-fed process. Moreover, optimization can be performed in a coupled manner, where both feeding modes are simultaneously allowed. This is illustrated in Figure 11.

Optimization was performed using an initial population of 10 individuals. Each design variable was encoded using a 5-bit binary representation. The crossover and mutation probabilities were set to 0.68 and 0.03, respectively. These parameters were selected as a compromise between computational efficiency and search capability. Since each fitness evaluation requires a complete simulation of the extrusion process, the population size was intentionally kept relatively small to reduce computational time while maintaining sufficient genetic diversity. The adopted crossover and mutation probabilities provided an appropriate balance between exploration of the search space and convergence toward the optimum solution. The optimization procedure was terminated when population stagnation was detected, i.e., when the best objective-function value remained unchanged for 20 consecutive generations. Additionally, a maximum of 100 generations was imposed to prevent excessive computational time. This stopping criterion was preferred to a fixed-generation approach because it allows optimization to continue only while the population evolves toward improved solutions.

One optimization run was performed for each weighting combination. Consequently, the present results demonstrate the operation of the optimization module but do not provide a statistical assessment of solution variability or robustness with respect to the initial population.

### 2.3. Process Scaling-Up

Process scaling-up was carried out by the GASES (Genetic Algorithm Screw Extrusion Scaling) program. The source of data for scaling-up is simulations carried out by the GSEM (Global Screw Extrusion Model) program.

The GASES scaling-up program, in co-operation with the GSEM simulation program, enables the extrusion process to be scaled up with various numbers of scaling variables and different scale-up criteria specified by single process parameters, e.g., specific energy consumption, solids transport rate, material plasticization rate, or pumping rate, as well as by process parameter profiles, e.g., the plasticization profile or temperature profile.

Summarizing, the scale-up criterion consists of five dimensionless objective function terms: three for scalar quantities and two for process profiles. Each term is expressed as an absolute value of relative deviation and contributes equally to the objective function value. The terms corresponding to the temperature and plasticization profiles are determined as the average absolute values of relative deviation calculated for n discretization points. By dividing these terms by n, the profiles do not exert a disproportionate influence on the objective function value simply due to the larger number of data points. As a result, each profile contributes a single, averaged term to the objective function, ensuring a balanced contribution of both scalar quantities and process profiles.

The variable coding procedure, chromosome representation, and response surface discretization applied in the GASES program are identical to those described previously for the GASEO optimization program (Section 2.2). A tournament selection method is applied during the scaling-up procedure to identify genotypes that provide the best agreement with the adopted scale-up criteria. In this approach, several genotypes are randomly selected from the population and compared with respect to the objective function value representing the scaling quality. The genotype characterized by the most favorable scaling result is transferred to the parent population and used in subsequent genetic operations. The selection procedure is repeated iteratively until the complete parent population is created. In the scaling-up procedure, the objective function usually represents the difference between the process characteristics of the reference extruder and those predicted for the target extruder. Consequently, genotypes producing smaller discrepancies with respect to the assumed scale-up criteria are preferentially selected for further evolution.

**Figure 12:**
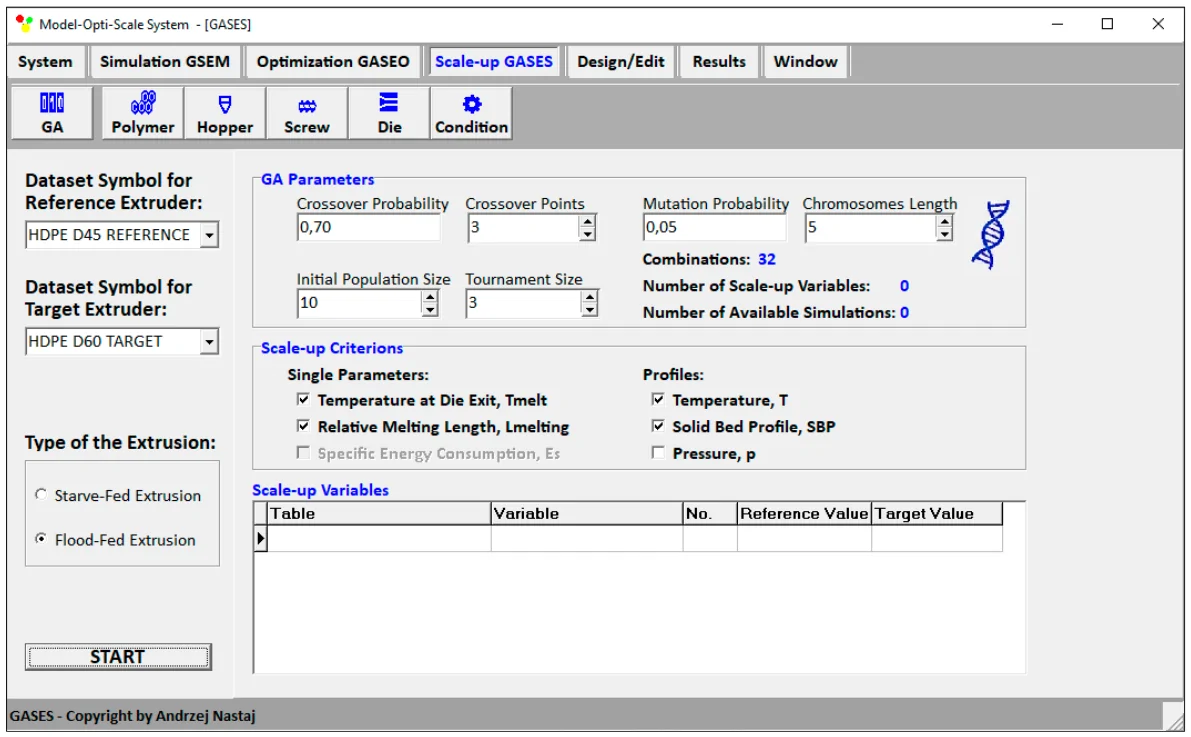
Parameters defining the scaling-up procedure.

The scaling-up procedure is defined by Genetic Algorithm variables, including the number of scaling variables, population size, chromosome length, crossover probability, number of crossover points, and mutation probability. Scaling-up can be performed for either the flood-fed process or starve-fed process. This is illustrated in Figure 12.

## 3. Process Design

### 3.1. Research Program

The problem of optimizing operating/geometry parameters of the single-screw process is presented to maximize process efficiency and minimize specific energy use. Optimization has been achieved in a unique way when both modes of feeding are allowed. This optimized process was then scaled up to increase process efficiency by enlarging the extruder diameter while keeping the length/diameter ratio constant. Scaling-up has been achieved using criteria comprising single parameters, including specific energy consumption, material temperature, and the plasticization rate, as well as criteria comprising functional parameters, such as temperature distribution and the plasticization profile. The simulation study program was developed to demonstrate the capabilities of the integrated Model-Opti-Scale (MOPS) system in modeling, optimization, and scaling the single-screw extrusion process. The system integrates three co-operating computational modules: GSEM (Global Screw Extrusion Model), GASEO (Genetic Algorithms Screw Extrusion Optimization), and GASES (Genetic Algorithms Screw Extrusion Scaling), enabling the comprehensive computer-aided design of plastics extrusion.

**Figure 13:**
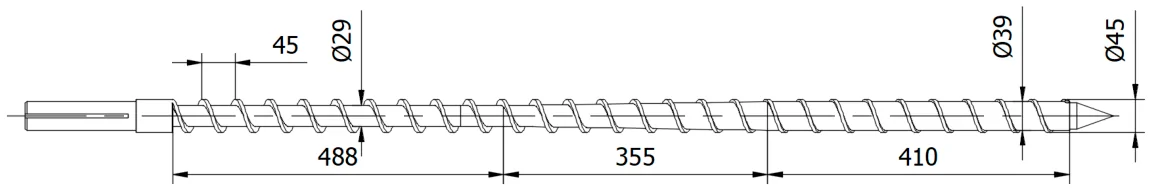
Geometry of a conventional screw.

Investigations were performed for the single-screw extrusion of high-density polyethylene (HDPE). A conventional, three-zone screw (Figure 13) of diameter *D* = 45 mm, a length/diameter ratio of *L*/*D* = 27, and a compression ratio of *CR* = 8/3 was used, and a cylindrical rod die of diameter *D* = 5 mm was applied. The aim of the study was to demonstrate the capabilities of the software in predicting the behavior of the extrusion process under various processing conditions and different material feeding modes.

The study was performed for both conventional gravity feeding (flood-fed extrusion) and metered feeding (starve-fed extrusion), which allows partial filling of the screw channel. The software enabled simulations over a wide range of processing parameters, including the screw speed *ν* = 20–80 rpm, variable lengths of the metering and compression (transition) regions of the screw, and different thermal processing conditions. This made it possible to analyze the influence of geometrical and operating parameters on the plasticization and material transport processes.

Within the modeling framework, the GSEM program enabled the determination of the main process characteristics, such as the pressure and temperature distributions, material plasticization profile, degree of screw filling, process throughput, and energy consumption. A particular feature of the program was its ability to automatically switch between flood-fed and starve-fed operating conditions while keeping the appropriate computational schemes.

Based on the simulation results, optimization of the extrusion process was performed using the GASEO program based on Genetic Algorithms. The study aimed to demonstrate the capabilities of the program in multi-objective optimization of the process while simultaneously considering technological and geometrical parameters. The optimization criteria were maximum process throughput, *G*_max_, and minimum specific energy consumption, *ε*_smin_. The optimized variables included the screw speed as well as the lengths of the metering and compression (transition) regions of the screw. The optimization studies made it possible to evaluate the effectiveness of the GASEO program in searching for optimal process parameters by using Genetic Algorithms and analyzing the influence of input parameters on the process behavior and objective function values.

In the final stage of the study, the GASES program was used to perform extrusion process scaling. The scaling investigations were intended to demonstrate the capabilities of the system in transferring process conditions from a reference extruder to a target extruder while keeping the similarity of process characteristics. The analysis considered criteria related to the material plasticization profile, temperature profile, specific energy consumption, and material flow characteristics.

Scaling procedures were carried out using multi-objective optimization methods, minimizing differences between the process characteristics obtained for the reference and target extruders. The program enabled scaling analysis for both flood-fed and starve-fed extrusion processes, allowing for evaluation of the influence of the feeding mode on keeping process similarity during changes in the scale of the extrusion process.

### 3.2. Optimization

Optimization was performed for single-screw extrusion of high-density polyethylene (HDPE) using a conventional screw (Figure 13), as in Section 3.1.

Flood-fed and starve-fed processes were investigated. Rigidex 6070EA (produced by BP Chemicals) was used, with a density ρ_S_ = 950 kg/m^3^ (solid), ρ_M_ = 720 kg/m^3^ (melt), and a Melt Flow Rate (MFR) = 7.6 g/10min (190 °C, 2.16 kg).

The polymer viscosity has been described by the Klein model:

(3) $$ln\eta =c_{0}+c_{1}ln\dot{\gamma }+c_{11}{ln}^{2}\dot{\gamma }+c_{12}\Theta ln\dot{\gamma }+c_{2}\Theta +c_{22}\Theta^{2}$$

where η represents the viscosity, $\dot{\gamma }$ represents the shear rate, Θ represents the temperature, and *c*_0_, c_1_, c_11_, *c*_12_, *c*_2_, c_22_ represent the model parameters (c_0_ = 10.92, c_1_ = −0.22, c_11_ = −0.037, c_12_ = 0.001, c_2_ = −0.023, c_22_ = 0.000021).

Optimization has been performed for both flood-fed and starve-fed processes to maximize process throughput $G_{max}$ and minimize specific energy consumption $\varepsilon_{s,min}$. The studies were conducted in two variants, with different weighting factors assigned to process throughput $\omega_{G}$ and specific energy consumption $\omega_{\varepsilon s}$. In the first variant, $\omega_{G}=0.9$ and $\omega_{\varepsilon s}=0.1$ were assumed, whereas in the second variant, $\omega_{G}=0.5$ and $\omega_{\varepsilon s}=0.5$. These weighting combinations $\left(\omega_{G},\;\omega_{\varepsilon s}\right)=\left(0.9,\;0.1\right)$ and $\left(\omega_{G},\;\omega_{\varepsilon s}\right)=(0.5,\;0.5)$ were selected to represent two illustrative technological priorities. The first case corresponds to a productivity-oriented strategy, in which throughput is assigned the dominant weight, whereas the second one represents a balanced compromise between throughput and specific energy consumption.

The optimized parameters included screw speed ν, cylinder temperatures *Θ*, and lengths *L* of the metering and compression (transition) regions, as shown in Table 1.

The screw speed range of 20–80 rpm refers to the general simulation examples, whereas the range of 40–120 rpm listed in Table 1 defines the admissible search range used in the optimization calculations. The throughput range of 20–40 kg/h applies to starve-fed operations, for which the material feeding rate is an imposed operating variable. Under flood-fed conditions, throughput is not independently prescribed but is calculated by the extrusion model as a process output.

The global objective function was defined as:

(4) $$\phi_{i}=\omega_{G}\cdot G_{{i\_}_{norm}}+\omega_{\varepsilon s}\cdot \varepsilon_{s\;i\_norm}$$

where the output variables (optimization criteria) have been normalized as:

(5) $$G_{i\_norm}=\frac{G_{i}-G_{min}}{G_{max}-G_{min}}$$

(6) $$\varepsilon_{s\;i\_norm}=\frac{\varepsilon_{s\;max}-\varepsilon_{s\;i}}{\varepsilon_{s\;max}-\varepsilon_{s\;min}}$$

where ϕ_i_ represents the global objective function, *G*_i_norm_ represents the flow rate, *ω*_G_ represents the weighting factor of flow rate, ε_s i_norm_ represents the specific energy use, ω_εs_ represents the weighting factor of specific energy use, *i* is the number of the next value from the variable set.

The results of optimizing for flood-fed/starve-fed extrusion are presented in Table 2.

The results of the simulations at optimal parameters for various weighting factors of optimization criteria are depicted in Figure 14, Figure 15, Figure 16, Figure 17, Figure 18, Figure 19, Figure 20 and Figure 21.

An analysis of the optimization results indicates the possibility of obtaining various optimal solutions, depending on the adopted design criterion. In the first variant of weights, i.e., $\omega_{G}=0.9$ and $\omega_{\varepsilon s}=0.1$, the solution is a configuration corresponding to the parameters $L_{m,opt}=45$ mm, $L_{t,opt}=351.29$ mm, and $\nu_{opt}=68.39$ rpm, for which the maximum process efficiency G = 22.65 kg/h was achieved with a moderate specific energy consumption of $\varepsilon_{s}=471.26$ kJ/kg. In the second variant of weights, i.e., $\omega_{G}=0.5$ and $\omega_{\varepsilon s}=0.5$, the optimal solution corresponds to the parameters $L_{m,opt}=45$ mm, $L_{t,opt}=307.74$ mm, and $\nu_{opt}=70.97$ rpm, and is characterized by a minimum specific energy consumption of $\varepsilon_{s}=413.22$ kJ/kg, but with a simultaneous reduction in process efficiency to G = 21.94 kg/h. These results confirm the multi-criteria nature of process optimization.

Summarizing, it is worth noting that changing from the flood-fed solution obtained for the productivity-oriented weighting combination to the starve-fed solution obtained for equal criterion weights resulted in a decrease in throughput from 22.65 to 21.94 kg/h, corresponding to approximately 3.1%. At the same time, specific energy consumption decreased from 471.26 to 413.22 kJ/kg, corresponding to approximately 12.3%. Thus, for the cases considered, a relatively small reduction in productivity was accompanied by a substantially larger relative improvement in energy efficiency.

Thus, it can be concluded that the system prefers the flood-fed regime when greater efficiency is the goal, while the starve-fed regime is preferred when lower energy consumption is the goal. The practical consequence is that when we use dosing (starve-fed regime) at a level close to the efficiency of gravitational feeding (flood-fed regime), i.e., slightly lower, we achieve a significant reduction in energy consumption.

The final results of process optimization can be seen on the screens of the GASEO program, which are depicted in Figure 20 and Figure 21. The optimization parameters are also shown, as well as the values of the optimal parameters.

### 3.3. Scaling-Up

Scaling-up was carried out for single-screw extrusion to enlarge the process output according to the scaling-up criteria determined by single process variables of specific energy use, plasticization rate, and melt temperature, and by process variable profiles of temperature and plasticization.

The research program included scaling-up from the reference extruder using a screw of diameter D = 45 mm to the target extruder using a screw of diameter D = 60 mm while keeping the length/diameter (L/D) ratio constant. Scaling up has been performed for both flood-fed and starve-fed extrusion, the process variables of which were determined from process optimization (Section 3.2). Screw geometry parameters of the target extruder for flood-fed extrusion and starve-fed extrusion are presented in Figure 22 and displayed in Table 3.

**Figure 14:**
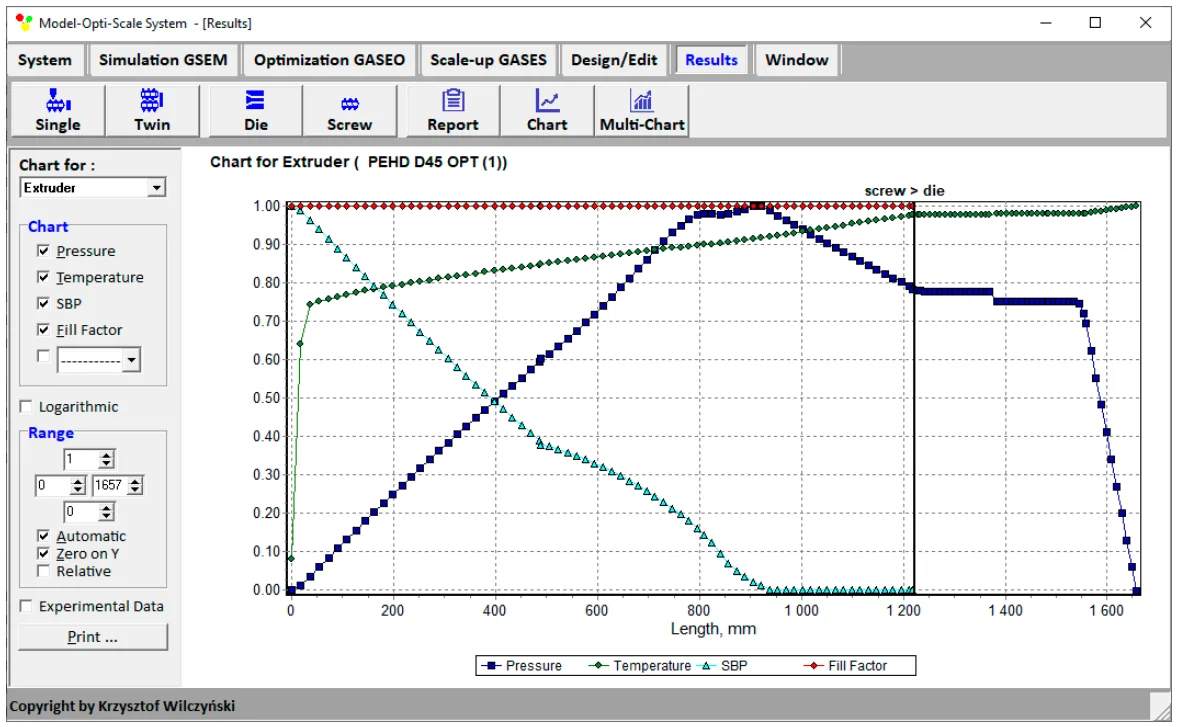
Extrusion characteristics: flood-fed process at weighting factors of optimization criteria ω_G_ = 0.9 and ω_εs_ = 0.1.

**Figure 15:**
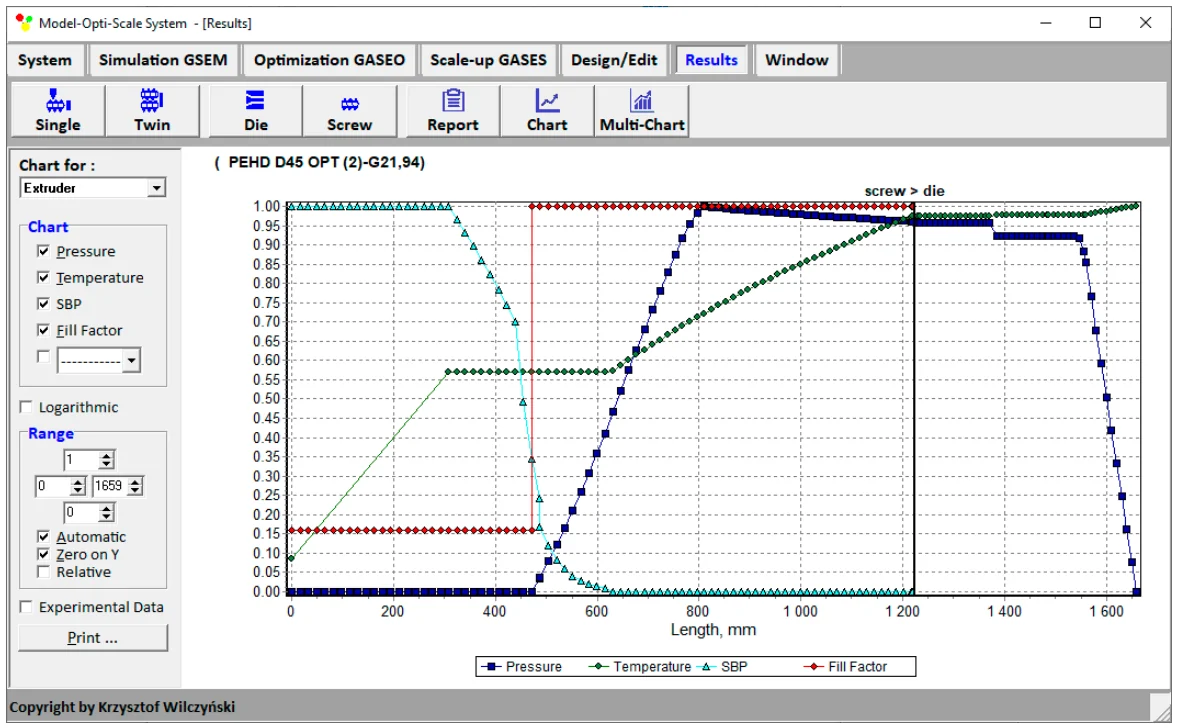
Extrusion characteristics: starve-fed process at weighting factors of optimization criteria ω_G_ = 0.5 and ω_εs_ = 0.5.

**Figure 20:**
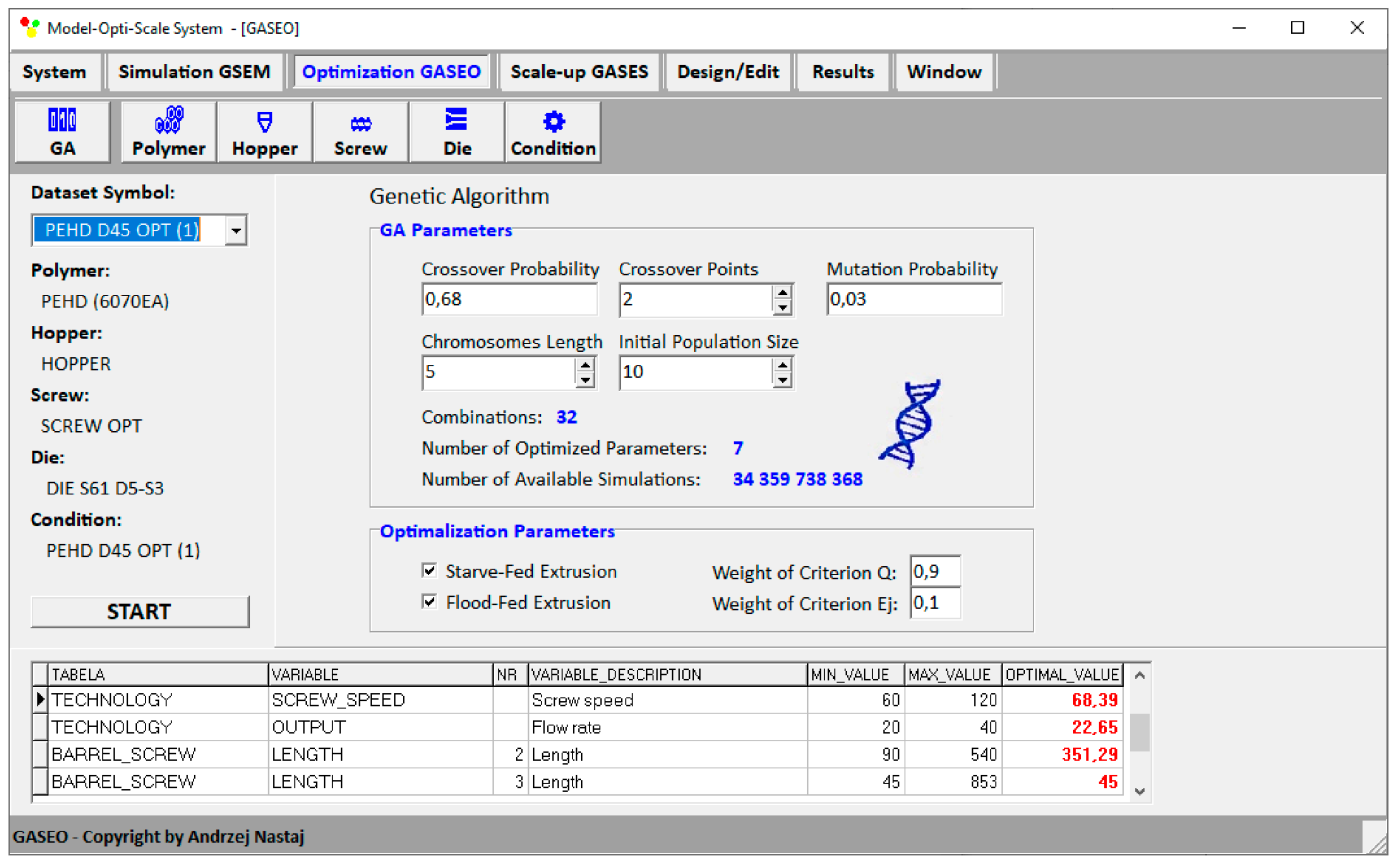
Optimization results: flood-fed process at weighting factors of optimization criteria ω_G_ = 0.9 and ω_εs_ = 0.1.

**Figure 21:**
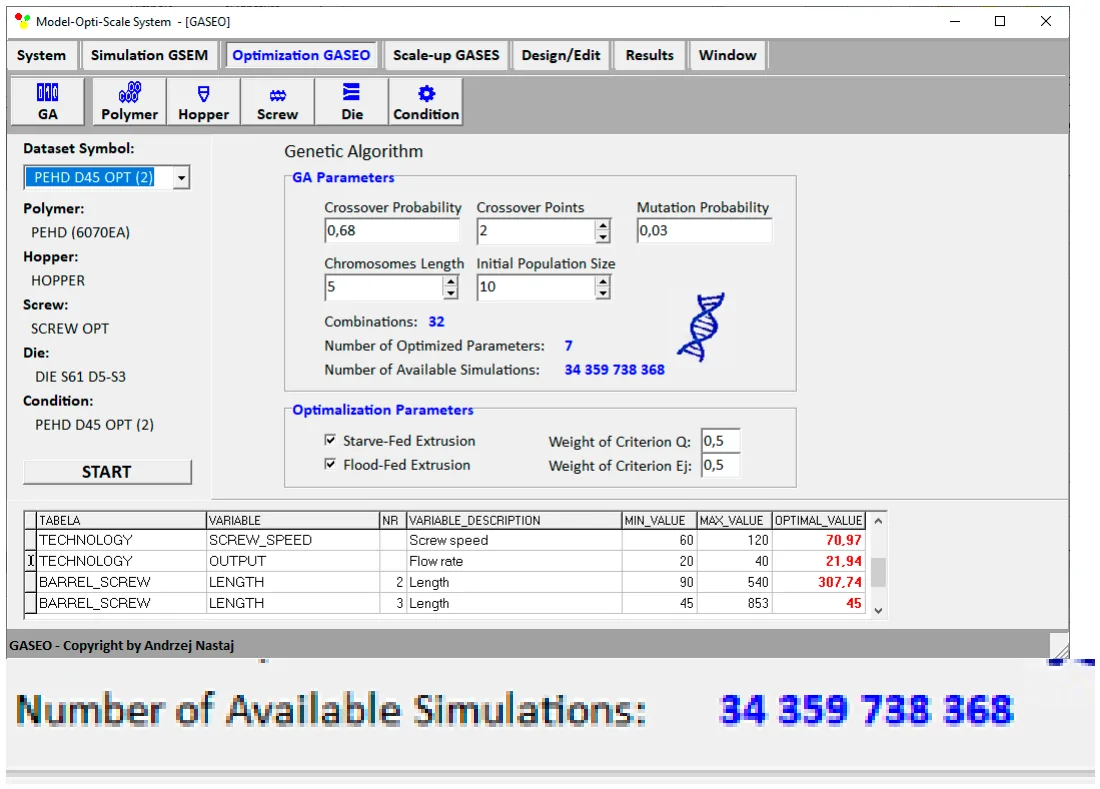
Optimization results: starve-fed process at weighting factors of optimization criteria ω_G_ = 0.5 and ω_εs_ = 0.5.

**Figure 22:**
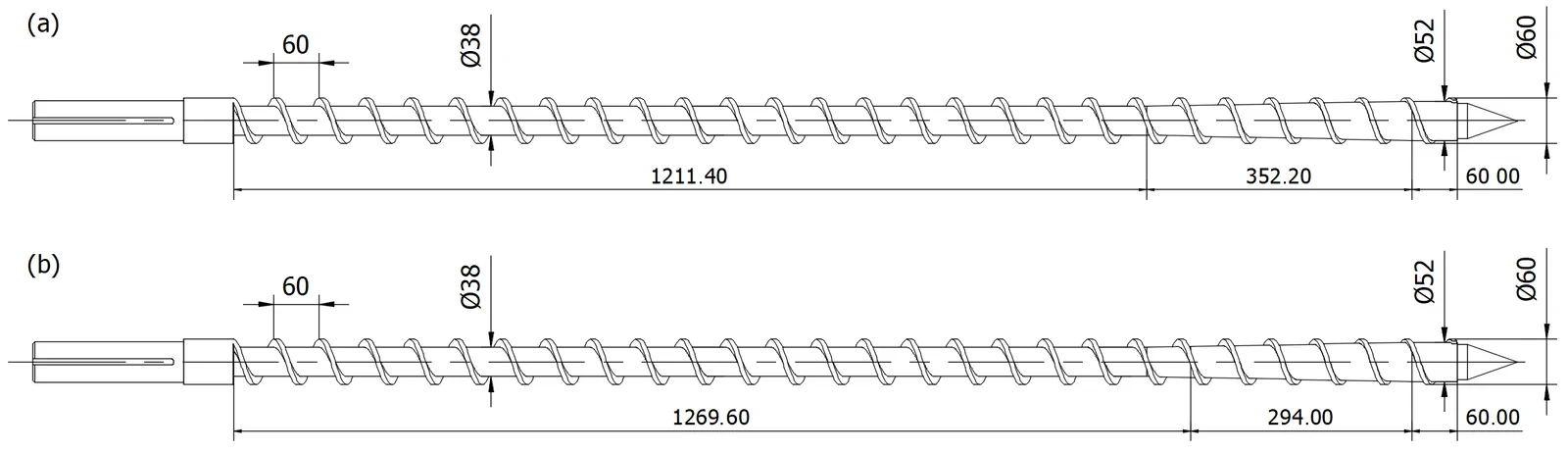
Geometry of the target extruder: (**a**) flood-fed process and (**b**) starve-fed process.

The results of the simulation for the reference extruder at optimal process variables are shown in Figure 14 and Figure 15.

Scaling-up was performed in the same range (as in process optimization) of input variables of screw speed *ν* = 40 ÷ 120 rpm and cylinder temperatures *Θ*_I_ = 150 °C, *Θ*_II_ = 180 ÷ 250 °C, *Θ*_III_ = 180 ÷ 250 °C, *Θ*_IV_ = 180 ÷ 250 °C. It was carried out according to the criteria of single parameters of specific energy use ε_s_, melt temperature Θ_melt_, and melting length *L*_melting_ (the ratio of the screw length needed for material plasticization to the total screw length), and according to the functional criteria of the temperature distribution and plasticization profile.

The global objective function was expressed as:

(7) $$\phi_{is}=\left|1-\frac{\varepsilon_{s\;A}}{\varepsilon_{s\;B_{i}}}\right|+\left|1-\frac{\Theta_{{melt}_{A}}}{\Theta_{{melt\;B}_{i}}}\right|+\left|1-\frac{L_{melting\;A}}{L_{melting\;B_{i}}}\right|+\frac{\sum_{k=1}^{n}\left|1-\frac{\Theta_{A\;k}}{\Theta_{B\;ik}}\right|}{n}+\frac{\sum_{k=1}^{n}\left|1-\frac{{SBP}_{A\;k}}{{SBP}_{B\;ik}}\right|}{n}$$

where ϕ*_i_* _s_ represents the global objective function, ε_s A_ represents the specific energy use in the reference extruder, ε_s B_ *_i_* represents the specific energy use in the target extruder, Θ_melt A_ represents the temperature at the die outlet in the reference extruder, Θ_melt B_ *_I_* represents the temperature at the die outlet in the target extruder, *L*_melting A_ represents the melting length in the reference extruder, *L*_melting B_ *_i_* represents the melting length in the target extruder, Θ_A_ represents the temperature in the reference extruder, Θ_B_ *_i_* represents the temperature in the target extruder, *SBP*_A_ represents the plasticization profile in the reference extruder, *SBP*_B_ *_i_* represents the plasticization profile in the target extruder, *i* represents the number of the next value from the variables set, and *n* represents the number of the next value in the profile.

**Figure 23:**
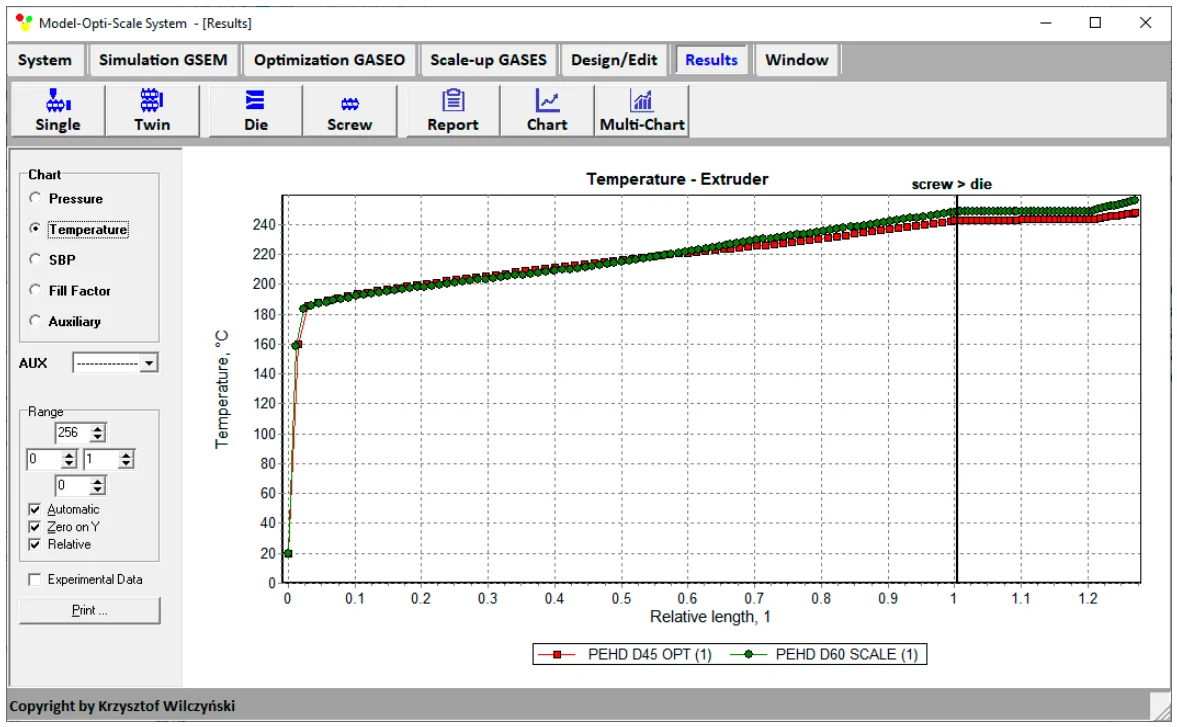
Temperature distribution for flood-fed extrusion. Red line—reference extruder; green line—target extruder.

**Figure 24:**
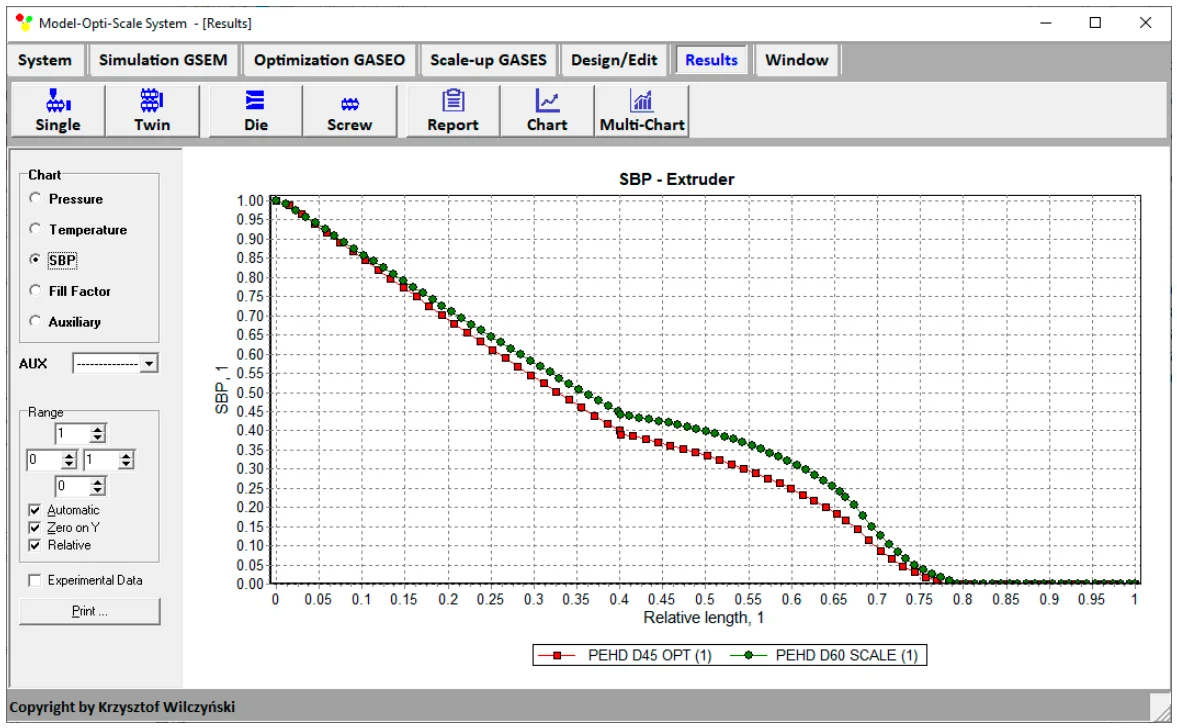
Solid bed profile (*SBP*) for flood-fed extrusion. Red line—reference extruder; green line—target extruder.

The scaling results for flood-fed extrusion are summarized in Table 4 and presented in Figure 23 and Figure 24. The smallest value of the objective function (Equation (7)), i.e., the minimum difference between the variables of the reference and target extrusion, was obtained at a screw speed of ν = 55.48 rpm for the cylinder temperatures: *Θ*_I_ = 150 °C, *Θ*_II_ = 180 °C, *Θ*_III_ = 198.06 °C, and *Θ*_IV_ = 204.84 °C. These data correspond to the process output variables of flow rate *G* = 32.72 kg/h, specific energy use ε_s_ = 469.05 kJ/kg, and melt temperature *Θ*_melt_ = 247.77 °C, as well as melting length *L*_melting_ = 0.784. The differences between the reference and target variables are small (Table 4). Therefore, it can be stated that these processes are similar in terms of the assumed criteria. The distributions of temperature and plasticization are also similar, as shown in Table 4 and Figure 23 and Figure 24. By enlarging the extrusion scale, substantial growth in the extrusion output was achieved (44.46%).

**Figure 25:**
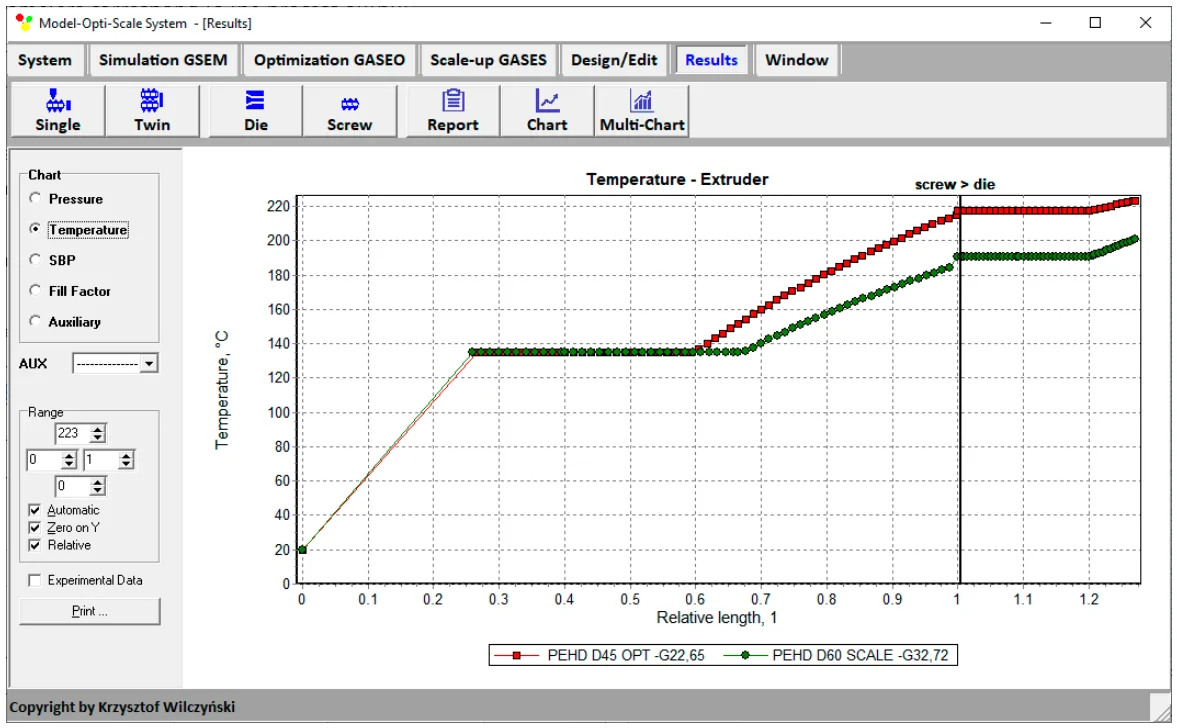
Temperature distribution for starve-fed extrusion. Red line—reference extruder; green line—target extruder.

**Figure 26:**
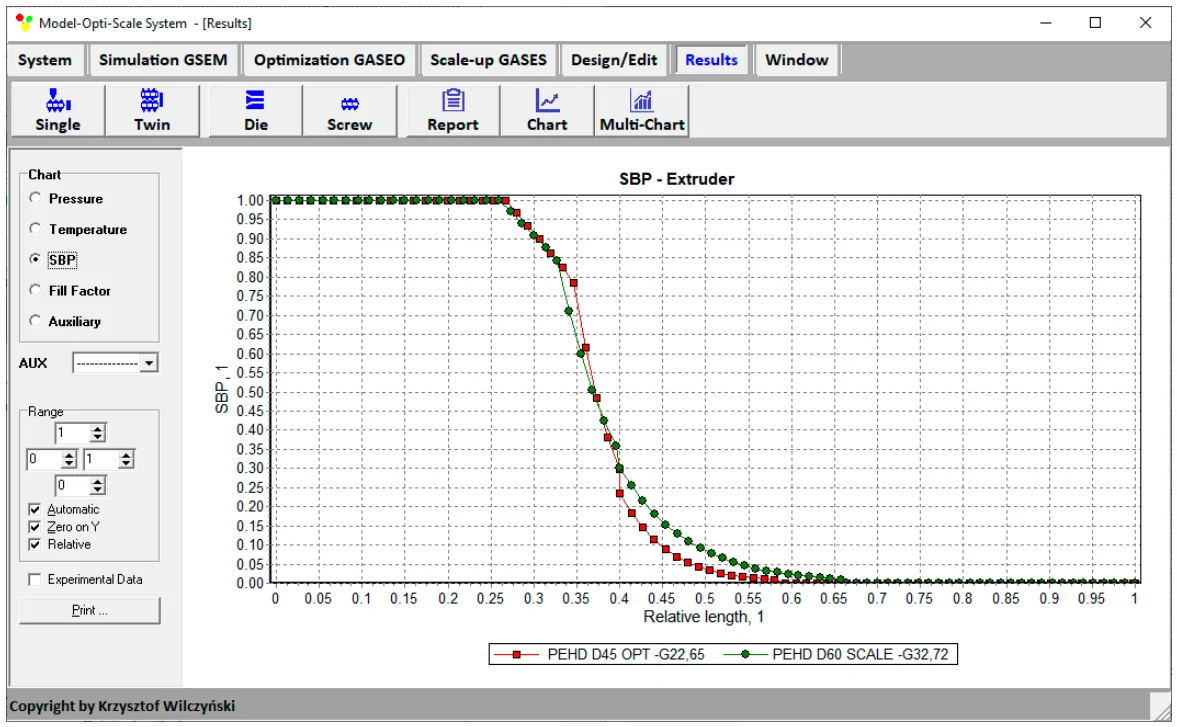
Solid bed profile (*SBP*) for starve-fed extrusion. Red line—reference extruder; green line—target extruder.

The scaling results for starve-fed extrusion are summarized in Table 5 and presented in Figure 25 and Figure 26. The smallest value of the objective function (Equation (7)), i.e., the minimum difference between the parameters of the reference and target extrusion, was obtained at a screw speed of ν = 57.14 rpm for the cylinder temperatures: *Θ*_I_ = 150 °C, *Θ*_II_ = 198.06 °C, *Θ*_III_ = 198.06 °C, and *Θ*_IV_ = 191.29 °C. These data correspond to the process output variables of flow rate *G* = 32.72 kg/h, specific energy use ε_s_ = 437.35 kJ/kg, and melt temperature *Θ*_melt_ = 216.98 °C, as well as melting length *L*_melting_ = 0.633. The differences between the reference variables and target variables are small (Table 5). Therefore, it can be stated that these processes are similar in terms of the assumed criteria. The distributions of temperature and plasticization are also similar and are shown in Table 5 and Figure 25 and Figure 26. By enlarging the extrusion scale, a substantial growth in extrusion output was achieved (49.13%).

## 4. Summary and Conclusions

This paper presents an integrated Model-Opti-Scale (MOPS) system developed for computer-aided design of single-screw plastics extrusion. The system integrates process modeling, optimization, and scaling-up within a single computational environment, enabling comprehensive analysis of the extrusion process under both flood-fed and starve-fed operating conditions.

As part of this work, the global extrusion model GSEM (Global Screw Extrusion Model) was developed and applied to simulate the material solid and melt conveying, as well as material plasticization in the screw/die system. The model enabled determination of the fundamental process characteristics, such as pressure and temperature profiles, material plasticization profile, screw filling profile, extrusion throughput, and energy consumption. The applied computational algorithms allowed for the analysis of the process both for fully filled screw channels and for partially filled conditions, characteristic of starve-fed extrusion.

Based on the simulation results, a multi-objective optimization of the extrusion process was carried out using the GASEO (Genetic Algorithms Screw Extrusion Optimization) program based on Genetic Algorithms. The investigations included analysis of the influence of technological and geometrical screw parameters, particularly screw rotational speed and metering/transition region lengths, on the extrusion process performance. The optimization criteria included maximizing process throughput and minimizing specific energy consumption. The obtained results confirmed the possibility of effectively identifying compromise solutions between process efficiency and system energy consumption.

For the weighting combinations and operating conditions considered in this study, the optimum corresponded to flood-fed operation when throughput was assigned the dominant weight. In contrast, starve-fed operation was selected when throughput and specific energy consumption were assigned equal weights.

The two weighting combinations $\left(\omega_{G},\omega_{\varepsilon_{s}})=(0.9,\;0.1\right)$ and $\left(\omega_{G},\;\omega_{\varepsilon s}\right)=(0.5,\;0.5)$ assumed in these studies should be regarded as illustrative cases demonstrating the multi-criteria optimization capabilities of the MOPS system. They do not provide an exhaustive characterization of the trade-off between throughput and specific energy consumption and should not be interpreted as defining a complete Pareto front.

The performed analyses demonstrated the extensive capabilities of the system for carrying out the comprehensive optimization studies that would be practically impossible to realize experimentally. Owing to the application of binary coding of process parameters and Genetic Algorithms, it was possible to analyze a large number of geometrical and technological configurations of the extrusion process while keeping a reasonable computational time.

The crucial novelty of this paper is the development of an integrated computational architecture in which the modeling, optimization, and scaling of the extrusion process are performed sequentially and in an interconnected manner. A particularly innovative element is the uniform treatment of flood-fed and starve-fed processes, the ability to seamlessly transition between these feeding methods, and the simultaneous optimization of both feeding methods. In this case, the choice of flood-fed or starve-fed is not imposed by the user but rather results from the optimization process. The resulting optimal process can then be directly scaled according to both scalar criteria and entire process profiles. Such a comprehensive combination of these functionalities has not been available in a single computer system so far.

In conclusion, the authors consider extending the holistic approach to modeling the polymer processing used in this work to the injection molding process. They are inspired by Donovan’s studies [130], according to which “the screw recharge in the injection molding is the transient plasticizing process, which gradually approached the equilibrium extrusion as the screw rotated. If screw rotation accounted for a large portion of the whole cycle time, plasticizing is similar to the extrusion, but if the screw rotation accounted for a small portion of the whole cycle time, plasticizing is significantly different”. As such, the authors believe it is worth the work.

## Figures and Tables

**Figure 16:**
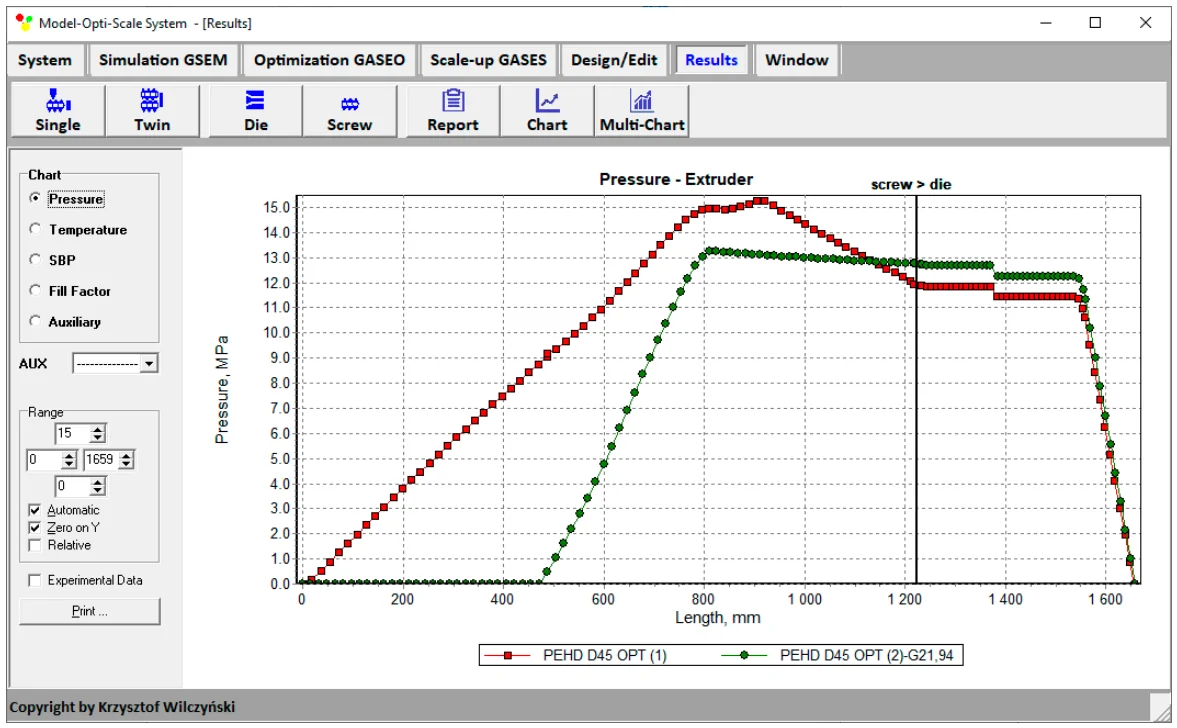
Pressure distributions at optimal parameters for various weighting factors of optimization criteria, ω_G_ and ω_εs_ (Table 2, results 1, 2). Red line—flood feeding; green line—starve feeding.

**Figure 17:**
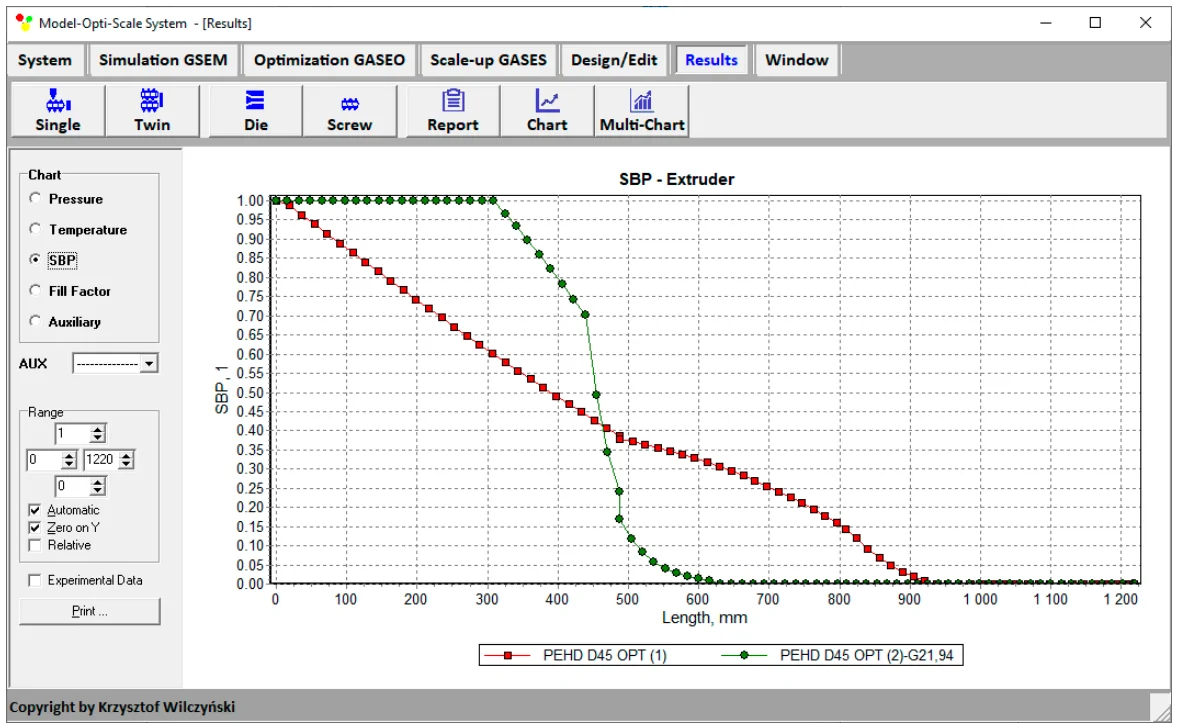
Solid bed profiles (SBP) at optimal parameters for various weighting factors of optimization criteria, ω_G_ and ω_εs_ (Table 2, results 1, 2). Red line—flood feeding; green line—starve feeding.

**Figure 18:**
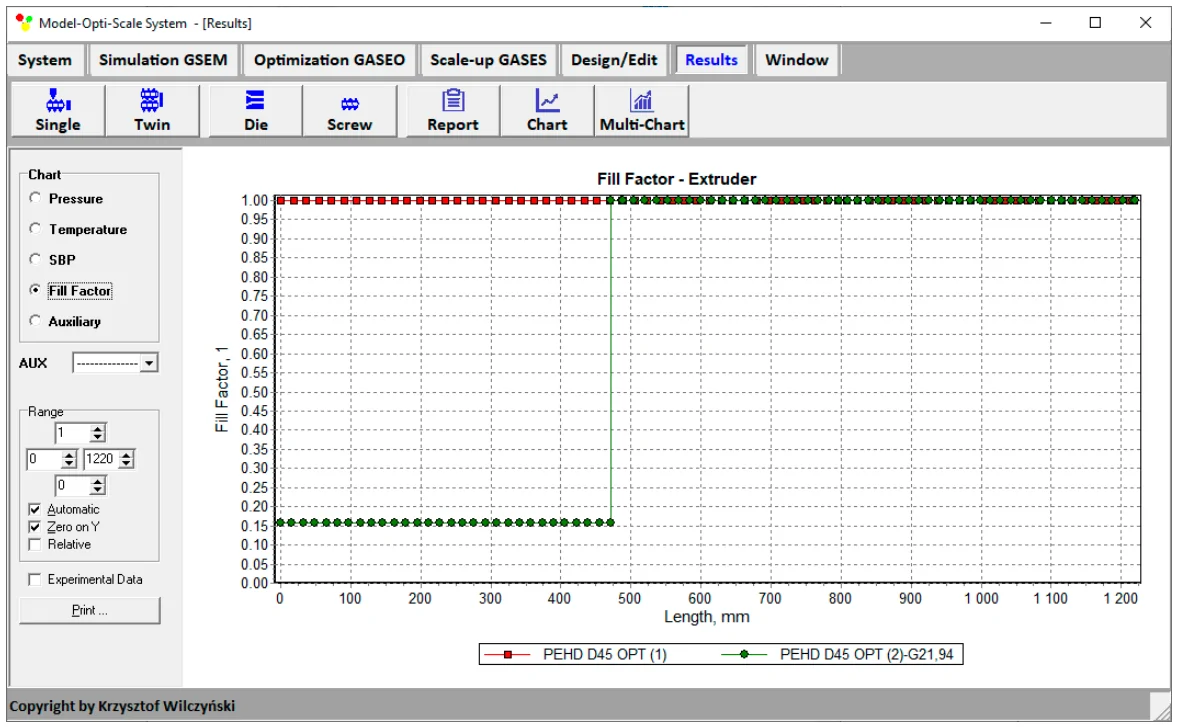
Filling factor distributions at optimal parameters for various weighting factors of optimization criteria, ω_G_ and ω_εs_ (Table 2, results 1, 2). Red line—flood feeding; green line—starve feeding.

**Figure 19:**
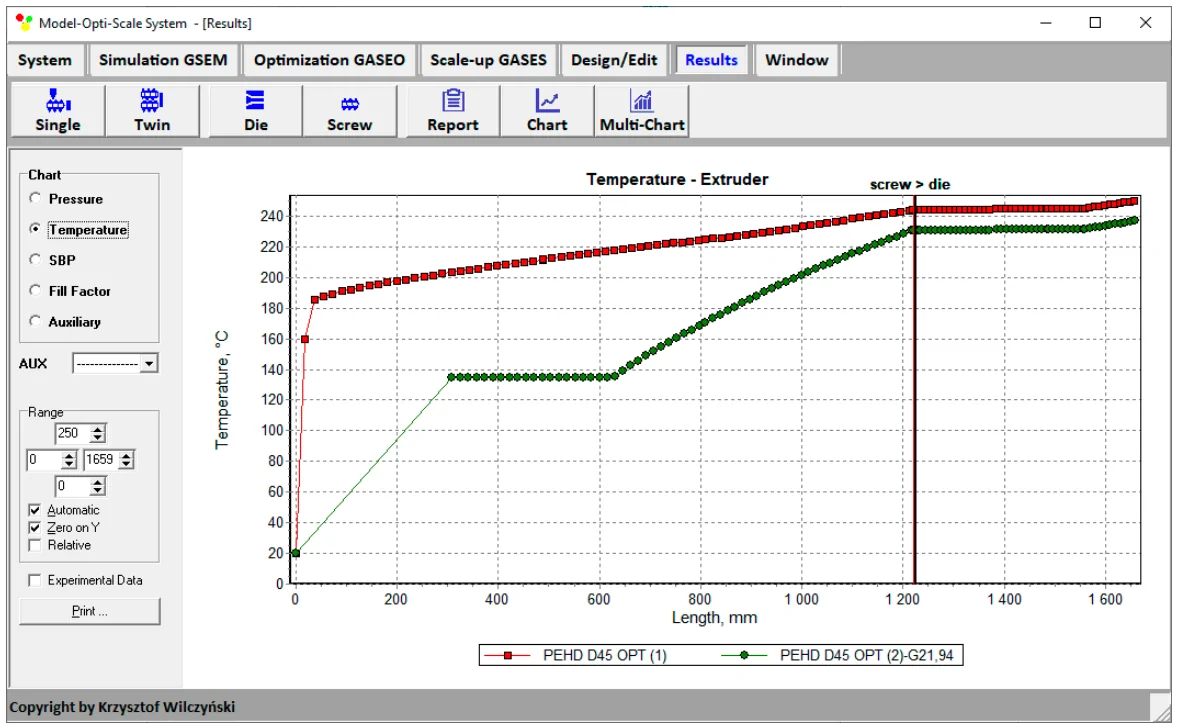
Temperature distributions at optimal parameters for various weighting factors of optimization criteria, ω_G_ and ω_εs_ (Table 2, results 1, 2). Red line—flood feeding; green line—starve feeding.

**Table 1:** Research program.

Parameter	Range	
	min	max
Screw speed ν, rpm	40	120
Length of screw metering region L_m_, mm	45	853
Length of screw transition region L_t_, mm	90	540
Cylinder temperature Θ_I_, °C	150	
Cylinder temperature Θ_II_, °C	180	250
Cylinder temperature Θ_III_, °C	180	250
Cylinder temperature Θ_IV_, °C	180	250
Flow rate G, kg/h	20	40

**Table 2:** Maxima of global functions at selected weighting factors of optimizing criteria.

	Weighting Factor of Criterion	
	ω_G_ = 0.9, ω_εs_ = 0.1	ω_G_ = 0.5, ω_εs_ = 0.5
No	1	2
Screw speed ν, rpm	68.39	70.97
Length of screw metering region L_m_, mm	45	45
Length of screw transition region L_t_, mm	351.29	307.74
Cylinder temperature Θ_II_, °C	193.55	240
Cylinder temperature Θ_III_, °C	180	240
Cylinder temperature Θ_IV_, °C	180	229.68
Flow rate G, kg/h	22.65	21.94
Specific energy use ε_s_, kJ/kg	471.26	413.22
Starve/Flood	Flood	Starve
Objective function ϕ_i_	0.9511	0.8642

**Table 3:** Screw geometry.

Screw Geometry		
	Reference	Target
Feed/transition/metering regions:		
- Flood-fed extrusion	20.19/5.87/1.00 turns	20.19/5.87/1.00 turns
- Starve-fed extrusion	21.16/4.90/1.00 turns	21.16/4.90/1.00 turns
Cylinder diameter, D_b_	45 mm	60 mm
Screw pitch	45 mm	60 mm
Channel depth in feed region, H_F_	8 mm	12 mm
Channel depth in metering region, H_M_	3 mm	4.5 mm
Flight width	5 mm	5 mm

**Table 4:** Scaling-up results of flood-fed extrusion.

Scaling-Up Results of Flood-Fed Extrusion			
	Extruder		
Single Parameters	Reference	Target	Difference
Specific energy consumption	471.26 kJ/kg	469.05 kJ/kg	0.47%
Melting length	0.756	0.784	3.70%
Melt temperature	256.13 °C	247.77 °C	3.38%
Extrusion output/Feeding flow rate	22.65 kg/h	32.72 kg/h	44.46%
**Profiles**			
Temperature distribution			
1.	20.00 °C	20.00 °C	0.00%
…	…	…	…
21.	201.64 °C	199.79 °C	4.42%
22.	202.32 °C	200.44 °C	4.78%
…	…	…	…
141.	255.22 °C	247.42 °C	3.27%
142.	256.13 °C	247.77 °C	3.38%
Solid bed profile			
1.	1.00	1.00	0.00%
…	…	…	…
21.	0.68	0.65	4.42%
22.	0.66	0.63	4.78%
…	…	…	…
73.	0.02	0.01	-
74.	0.01	0.00	-
75.	0.00	0.00	-

**Table 5:** Scaling-up results of starve-fed extrusion.

Scaling-Up Results of Starve-Fed Extrusion			
	Extruder		
Single Parameters	Reference	Target	Difference
Specific energy use	413.22 kJ/kg	437.35 kJ/kg	5.84%
Melting length	0.623	0.633	1.61%
Melt temperature	236.79 °C	216.98 °C	8.37%
Extrusion output/Feeding flow rate	21.94 kg/h	32.72 kg/h	49.13%
**Profiles**			
Temperature distribution			
1.	20.00 °C	20.00 °C	0.00%
…	…	…	…
39.	135.00 °C	135.00 °C	0.00%
40.	135.00 °C	135.38 °C	0.28%
…	…	…	…
125.	234.45 °C	216.32 °C	7.73%
126.	236.79 °C	216.98 °C	7.59%
Solid bed profile			
1.	1.00	1.00	0.00%
…	…	…	…
21.	0.95	0.84	11.55%
22.	0.91	0.80	11.76%
…	…	…	…
39.	0.01	0.13	-
40.	0.00	0.09	-
41.	0.00	0.04	-

## Data Availability

The original contributions presented in this study are included in the article. Further inquiries can be directed to the corresponding author.
